# The potential impact of Brexit and immigration policies on the GP workforce in England: a cross-sectional observational study of GP qualification region and the characteristics of the areas and population they served in September 2016

**DOI:** 10.1186/s12916-017-0953-y

**Published:** 2017-11-16

**Authors:** Aneez Esmail, Maria Panagioti, Evangelos Kontopantelis

**Affiliations:** 1 NIHR School for Primary Care Research, University of Manchester, Manchester, UK; 2 Division of Population Health, Health Services Research & Primary Care, University of Manchester, 7th floor Williamson Building, Manchester, UK; 3 Division of Informatics, Imaging & Data Sciences, University of Manchester, Manchester, UK

**Keywords:** Medical immigration, General practitioners, Primary care, Doctor country of qualification, Deprivation, Workforce, Brexit, European Economic Area (EEA), Englandx

## Abstract

**Background:**

The UK is dependent on international doctors, with a greater proportion of non-UK qualified doctors working in its universal health care system than in any other European country, except Ireland and Norway. The terms of the UK exit from the European Union can reduce the ability of European Economic Area (EEA) qualified doctors to work in the UK, while new visa requirements will significantly restrict the influx of non-EEA doctors. We aimed to explore the implications of policy restrictions on immigration, by regionally and spatially describing the characteristics of general practitioners (GPs) by region of medical qualification and the characteristics of the populations they serve.

**Methods:**

This is a cross-sectional study on 37,792 of 41,865 GPs in England, as of 30 September 2016. The study involved age, sex, full-time equivalent (FTE), country and region of qualification and geography (organisational regions) of individual GPs. Additionally at the practice and geography levels, we studied patient list size by age groups, average patient location deprivation, the overall morbidity as measured by the Quality and Outcomes Framework (QOF) and the average payment made to primary care per patient.

**Results:**

Non-UK qualified GPs comprised 21.1% of the total numbers of GPs, with the largest percentage observed in East England (29.8%). Compared to UK qualified GPs, EEA and elsewhere qualified GPs had higher FTE (medians were 0.80, 0.89 and 0.93, respectively) and worked in practices with higher median patient location deprivation (18.3, 22.5 and 25.2, respectively). Practices with high percentages of EEA and elsewhere qualified GPs served patients who resided in more deprived areas, had lower GP-to-patient ratios and lower GP-to-cumulative QOF register ratios. A decrease in pay as the percentage of elsewhere qualified GPs increased was observed; a 10% increase in elsewhere qualified GPs was linked to a £1 decrease (95% confidence interval 0.5–1.4) in average pay per patient.

**Conclusions:**

A large percentage of the UK general practice workforce consists of non-UK qualified GPs who work longer hours, are older and serve a larger number of patients in more deprived areas. Following Brexit, difficulties in replacing this valuable workforce will primarily threaten the care delivery in deprived areas.

**Electronic supplementary material:**

The online version of this article (doi:10.1186/s12916-017-0953-y) contains supplementary material, which is available to authorized users.

## Background

Current General Medical Council (GMC) estimates suggest that 36% of doctors working in the UK obtained their primary medical qualification outside the UK. Twenty-two percent of these are general practitioners (GPs) [1]. These international medical graduates (IMGs) provide a valuable service to the National Health Service (NHS), often working in areas that are unpopular with British graduates — primary care in inner city areas, ex-mining communities and in specialties like psychiatry and geriatrics [2]. It is surprising that the world’s fifth richest country by gross domestic product (GDP) [3] is so dependent on international doctors. In fact, the UK has a greater proportion of non-UK qualified doctors working in its universal health care system than any other European country, with the exception of Ireland and Norway [4].

The UK’s continued dependence on doctors who have qualified outside the UK may amplify the current NHS crisis, due to several factors. The terms of the UK exit from the European Union could potentially reduce the ability of EEA qualified doctors to work in the UK. Furthermore, new visa requirements with the removal of the permit free training visa and the introduction of the Tier 2 visa (which can only be issued if no UK or EU resident with ‘leave to remain’ satisfied the person specification for a post) significantly restricts the ability of non-EEA nationals to work in the NHS [5].

Additional initiatives such as the Medical Training Initiative, which is administered by the Academy of Medical Royal Colleges, offer a time-limited 2-year Tier 5 visa ostensibly to provide training for overseas graduates but they also explicitly state that the visa can be used to employ overseas graduates in under-subscribed and of-need areas for hospital trusts [6]. The scheme offers the doctors recruited the ability to practice in the UK without the requirements of the Professional and Linguistic Assessment Board (PLAB) test. The aim is that the candidate returns home after 2 years of training. It does not provide a mechanism for doctors to continue to remain in the UK after this period. The scheme is currently being used to recruit IMGs to shortage specialties such as accident and emergency (A&E) but can realistically only apply to hospital training posts because of the training requirements for general practice. Further unintended consequences of the Brexit vote and the negative political climate around immigration may also make the UK a less appealing destination for medical migrants, compounding the problems outlined above.

The problems of GP workforce recruitment are likely to be exacerbated by these changes because there is an unequal distribution of GPs across the country, with areas of high deprivation, where health care needs are greater, having fewer GPs per head than the UK average [7]. In addition, GPs tend to be older in deprived areas [8], while evidence from surveys suggests that 54% of GPs over the age of 50 are intending to quit direct patient care within 5 years [9], with the rate increasing to approximately 80% for those aged 55 or older [10]. There is anecdotal evidence that these areas of high deprivation have a higher proportion of IMGs who also tend to be older (over 50) and are therefore more likely to retire in the next decade. We first described this scenario in a paper published in 1999 [11]. The availability of more extensive workforce data may allow further detailed analysis of health care needs and their relationship to workforce characteristics based on the distribution of GPs, their country of qualification and the workforce demands that might be affected by changes in immigration policy.

In this paper we aimed to investigate the geographical location, distribution and serving populations of non-UK qualified GPs, with a particular focus on EEA and elsewhere qualified GPs. There is currently no up-to-date information on the distribution of GPs by country of qualification and their demographics. It is our assertion that there will be areas of the country which will be more adversely affected by changes in immigration policy post-Brexit. It is therefore important that commissioners, Health Education England (HEE) and the wider NHS are aware of the significant problems this might have on future workforce planning needs and the provision of primary care services. The underlying hypothesis was that in the EEA and elsewhere (not in the UK or the EEA) qualified GPs serve more deprived populations with more health needs, while often provided with fewer resources. We also investigated the characteristics of GPs and their geographical distribution, by country of qualification.

## Methods

### Data

We used a range of sources of administrative and spatial data. The core dataset that contains primary care workforce information is provided by NHS Digital, and we obtained data as of 30 September 2016, published on 29 March 2017 [12]. At the practice level, information is available on geography (Clinical Commissioning Group (CCG) and NHS region), patient list size by age groups and also numbers and full-time equivalent (FTE) for GPs, by country and region of qualification. At the individual level, the same geographical information is available, as well as staff type (e.g. GP, nurse, administrator), role (e.g. GP partner, junior doctor), country and region of qualification for GPs only, age, sex and FTE. Individual records are not linked to practices to protect anonymity [13].

Additional information at the practice level included the average deprivation of patients, the overall morbidity as measured by the QOF and the average payment made to primary care per patient. NHS payments to general practice for the financial year 2015/2016 and for the whole of England [14] were used to calculate average pay per patient minus prescribing and dispensing fee payments.

Deprivation was quantified with the 2015 release of the Index of Multiple Deprivation (IMD), a complete aggregate measure which is widely used to quantify deprivation and affluence [15]. The measure quantifies relative deprivation across the following seven domains: income, employment, education and skills, health and disability, crime, barriers to housing and services, and living environment. Deprivation scores are calculated and assigned to very low UK geographical units (Lower Super Output Areas), and the overall IMD is calculated as a weighted mean across the seven domains, with income and employment deprivation given the largest weight (22.5% each), followed by health and education deprivation (13.5% each), and with the other three domains given equal weights (9.3%). To calculate the average deprivation levels of the practice population, rather than the practice location, we made use of a dataset linking practice populations to low geographies [16], allowing us to calculate a weighted average of deprivation for each practice.

To quantify overall morbidity at the practice level, we used 2015/2016 data from a national primary care pay-for-performance programme, the QOF [17]. The programme has underpinned high quality of recording in primary care [18], and under its umbrella, recording, management and treatment of a large number of clinical domains was financially and reputationally incentivised. In 2015/2016, there were 21 incentivised domains: Atrial Fibrillation, Asthma, Cancer, Cardiovascular Disease Primary Prevention, Coronary Heart Disease, Chronic Kidney Disease (for those aged 18 or older), Chronic Obstructive Pulmonary Disease, Dementia, Depression (18 or older), Diabetes both types (17 or older), Epilepsy (18 or older), Heart Failure, Hypertension, Learning Disability, Severe Mental Illness, Obesity (18 or older), Osteoporosis (50 or older), Peripheral Artery Disease, Palliative Care, Rheumatoid Arthritis (16 or older), Stroke. For each of the practices participating in the QOF, covering more than 99% of all registered patients [19], we calculated the total sum of all condition registers, a cumulative QOF register.

Finally, 2016 spatial coordinates for NHS organisational units were obtained from the Office for National Statistics (ONS) Open Geography portal [20]. We focused on two organisational levels, the lower CCGs with 209 units, and the higher NHS regions with 14 units.

### Analyses

For all aspects of data manipulation and analysis we used Stata v14.1. Whenever medians are reported, we also report the 25th and 75th centiles. Spatial maps were plotted using the *spmap* command [21]. An alpha level of 5% was used throughout.

We quantified the characteristics of GPs for the whole of England and for each of the 14 NHS regions in 2016. For each region and overall, we estimate and report the following individual aggregates: number, percentage of males, median age and median FTE. All individual-level aggregates are reported overall by GP country of qualification. We also report practice-level aggregates on median number and FTE (overall only) per 10,000 patients, per 1000 patients aged 75 or older and per 10,000 counts on the cumulative QOF register. Finally, and also at the practice level but overall and by GP country of qualification, we present the median residence location deprivation of the average practice patient and median of the average pay per patient (minus prescription and dispensing costs). For deprivation, we first calculated the weighted deprivation mean within each practice, and next we estimated its weighted median (weighted by the practice list size for overall estimates or by the product of the list size and the percentage of GPs qualified from each region for qualification regions). The process was similar for pay, the only difference being the first step where we used the average pay per patient in the practice. This weighting approach allowed us to estimate patient deprivation and pay medians by GP country of qualification (on numbers, which were available as country of qualification aggregates at the practice level, when FTE was not).

In a second approach, we quantified the characteristics of practices (median FTE per 10,000 patients, 1000 patients aged 75 or older and 10,000 counts on the cumulative QOF register; also average pay and patient residence location deprivation) at different levels of presence of overseas qualified GPs: 0%; above 0% and up to 20%; above 20% and up to 40%; and above 40%. To evaluate if pay differed for various strata of EEA and elsewhere qualification more robustly, we performed multiple linear regressions at the practice level, associating average pay to percentage of EEA, percentage of elsewhere and percentage of EEA or elsewhere qualification, adjusted for the percentage of patients aged 75 or older and the cumulative QOF register (i.e. adjusting for proxies of health need).

Spatial graphs at the CCG level with additional information on NHS regions were plotted for various variables of interest for GPs, overall and by region of qualification: number and percentage aged 55 or older (FTE weighted), cumulative FTE, mean age, FTE per 10,000 patients and FTE per 10,000 counts on the cumulative QOF register. The primary aim of these graphs was to identify areas more dependent on overseas qualified GPs.

## Results

Characteristics for GPs, by region of qualification, are provided in Table 1 for 92.4% of the general practices that submitted data. Elsewhere and EEA qualified GPs make up 21.1% of the workforce of 37,792 GPs for which individual-level information was available. The median GP age was 42, 48 and 46 for UK, EEA and elsewhere qualified, respectively. Median FTE for EEA and elsewhere qualified GPs was 0.89 and 0.93, respectively, compared to 0.80 for UK qualified. Across general practices, the median of the average patient location deprivation varied by GP region of qualification: 18.3 for UK (52nd centile of the IMD distribution across all low geographical regions for England), 22.5 for EEA (62nd centile) and 25.2 for elsewhere (67th centile). The general practice median of the average pay per patient (minus prescription and dispension costs) was £133 for UK qualified GPs, £132 for EEA qualified and £129 for elsewhere qualified. In Additional file 1 we also present this information for each of the 14 NHS regions (Additional file 1: Tables S1 and S2). The largest percentage of non-UK qualified GPs was observed in East England (29.8%) and the lowest in the South West (7.6%). The greatest variability in terms of average payment was seen in the East, with medians of £145 (UK qualified), £134 (EEA) and £126 (elsewhere). Average patient deprivation was consistently higher for EEA and elsewhere qualified GPs, across all regions.

**Table 1:** Individual and practice-level characteristics for the whole of England

Variable name	Qualification	All of England
Individual-level aggregates		
Number of GPs (%)	All	37,792 (100.0%)
	UK	28,636 (75.8%)
	EEA	1535 (4.1%)
	Elsewhere	6419 (17.0%)
	Unknown	1202 (3.2%)
Percentage male GPs	All	45.6
	UK	43.4
	EEA	50.2
	Elsewhere	54.9
	Unknown	43.3
Median GP age (25th, 75th centiles)	All	43 (35, 52)
	UK	42 (34, 52)
	EEA	48 (40, 54)
	Elsewhere	46 (40, 56)
	Unknown	40 (33, 49)
Median GP FTE (25th, 75th centiles)	All	0.85 (0.60, 1.00)
	UK	0.80 (0.60, 1.00)
	EEA	0.89 (0.64, 1.07)
	Elsewhere	0.93 (0.64, 1.04)
	Unknown	0.80 (0.53, 1.00)
Per 10,000 patients		
Median number of GPs (25th,75th centiles)	All	6.7 (5.2, 8.4)
Median FTE of GPs (25th,75th centiles)	All	5.2 (4.1, 6.4)
Per 1000 patients aged 75 or older		
Median number of GPs (25th,75th centiles)	All	8.6 (6.3, 12.6)
Median FTE of GPs (25th,75th centiles)	All	6.8 (5.1, 9.6)
Per 10,000 counts on the cumulative QOF register^a^		
Median number of GPs (25th,75th centiles)	All	11.3 (8.6, 15.0)
Median FTE of GPs (25th,75th centiles)	All	8.9 (6.9, 11.3)
Residence overall deprivation of the average patient^b,c^		
Median (25th, 75th centiles)	All	19.7 (13.0, 29.0)
	UK	18.3 (12.2, 27.0)
	EEA	22.5 (14.9, 30.5)
	Elsewhere	25.2 (17.0, 34.1)
	Unknown	22.4 (13.6, 33.2)
Average pay per patient^b,d^		
Median (25th, 75th centiles)	All	132 (120, 150)
	UK	133 (120, 152)
	EEA	132 (120, 150)
	Elsewhere	129 (117, 146)
	Unknown	131 (120, 145)

A break-down across the 14 NHS regions is provided in Additional file 1

^a^Twenty-one clinical domain registers for 2015/2016: Atrial Fibrillation, Asthma, Cancer, Cardiovascular Disease Primary Prevention, Coronary Heart Disease, Chronic Kidney Disease (for those aged 18 or older), Chronic Obstructive Pulmonary Disease, Dementia, Depression (18 or older), Diabetes both types (17 or older), Epilepsy (18 or older), Heart Failure, Hypertension, Learning Disability, Severe Mental Illness, Obesity (18 or older), Osteoporosis (50 or older), Peripheral Artery Disease, Palliative Care, Rheumatoid Arthritis (16 or older) and Stroke

^b^Weighted on list size for all qualified GPs; the product of list size and the percentage of the respective GP group within the practice (on number rather than FTE), for UK, EEA and elsewhere qualified

^c^Index of Multiple Deprivation, details available in the 2015 technical report of the English Indices of Deprivation [15]

^d^Minus prescription and dispensing costs

The tabulation by percentage of GPs qualified in the EEA or elsewhere is presented in Table 2. Practices with high percentages of EEA and elsewhere qualified GPs tend to have patients who reside in more deprived areas, have lower GP-to-patient ratios and have lower GP-to-cumulative QOF register ratios. In other words, practices with more EEA and elsewhere qualified GPs, on average, deal with more deprived patients of poorer health and have fewer GPs relative to the health needs (as quantified by the QOF). For older patients the picture differs for EEA and elsewhere qualified GPs, with a lower GP-to-older patients ratio for the former, and a higher one for the latter. Regarding average pay, the picture was inconsistent for EEA qualification (largely due to small practice numbers with high percentages of EEA qualified GPs), but we observed a decrease in pay as the percentage of elsewhere qualified GPs increased.

**Table 2:** Characteristics of practices at various levels of European Economic Area (EEA) and elsewhere qualified participation

		Qualified in the EEA				Qualified elsewhere			
	All	None	(0%, 20%]	(20%, 40%]	40% or more	None	(0%, 20%]	(20%, 40%]	40% or more
Number of practices	6477	5284	689	331	173	3055	973	930	1519
Median pay per patient (£)	133.0 (119.7, 153.1)	132.8 (119.4, 153.1)	134.4 (121.0, 152.8)	132.5 (121.3, 151.2)	134.2 (122.1, 159.1)	135.0 (121.5, 158.3)	134.2 (122.8, 152.5)	132.2 (118.5, 152.2)	128.2 (115.7, 146.6)
Median residence IMD of average practice patient	21.4 (13.7, 31.3)	21.2 (13.6, 31.5)	19.6 (13.0, 28.2)	24.1 (15.4, 31.8)	26.7 (19.5, 34.9)	18.3 (12.3, 27.9)	18.1 (12.0, 26.5)	22.6 (15.5, 32.5)	28.8 (20.5, 37.0)
*Median FTE of GPs in these practices*									
Per 10,000 patients	5.2 (4.1, 6.4)	5.2 (4.1, 6.4)	5.6 (4.7, 6.8)	5.0 (3.9, 6.0)	4.2 (3.1, 5.1)	5.3 (4.2, 6.6)	5.7 (4.8, 6.9)	5.1 (4.2, 6.1)	4.6 (3.6, 5.7)
Per 1000 patients aged 75 or older	6.8 (5.1, 9.6)	6.8 (5.0, 9.6)	7.2 (5.5, 9.6)	6.5 (4.9, 9.2)	6.3 (4.5, 10.2)	6.4 (4.9, 8.9)	7.1 (5.4, 9.4)	7.0 (5.3, 10.1)	7.4 (5.2, 11.1)
Per 10,000 counts on the cumulative QOF register	8.9 (6.9, 11.3)	8.8 (6.9, 11.2)	9.7 (7.7, 12.6)	8.2 (6.8, 10.7)	7.1 (5.5, 9.6)	9.0 (7.1, 11.4)	9.7 (7.8, 12.3)	8.7 (7.0, 10.9)	8.0 (6.2, 10.4)

A total of 3508 GPs (9.28%) were not linked to practices and could not be included in this table. Non-linkage varied greatly across NHS regions: London = 1160, Yorkshire and Humber = 2, Cumbria and North East = 3, Cheshire and Merseyside = 568, North Midlands = 12, Central Midlands = 1, East = 837, South West = 8, South East = 8, South Central = 293, Greater Manchester = 395, Unknown = 221)

The characteristics were calculated on numbers of GPs, not FTE (FTE data by country of qualification was not available at the practice level)

Complementing the tabulation with the multiple linear regression results, we found that pay was not associated with percentage of EEA qualified GPs in the practice, when adjusting for older patients and the cumulative QOF register (the effect was very small and not statistically significant). However, there was a modest adjusted association between pay and the percentage of elsewhere qualified GPs in the practice, with a 10% increase in elsewhere qualified GPs linked to a £1 decrease (95% confidence interval 0.5–1.4) in average pay per patient.

**Fig. 1:**
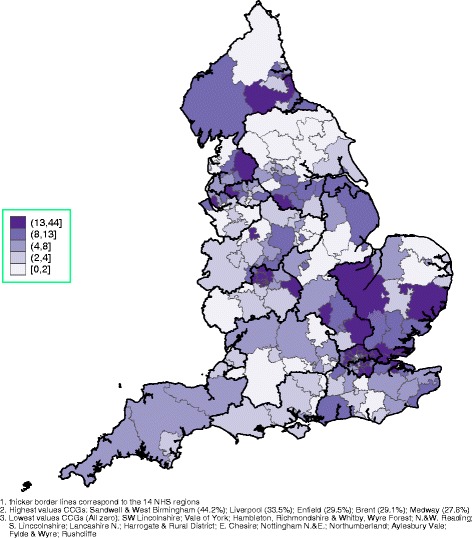
Spatial map at the CCG level, September 2016: cumulative FTE of GPs aged 55 or older, EEA or elsewhere qualified^1,^ ^2,^ ^3^

**Fig. 2:**
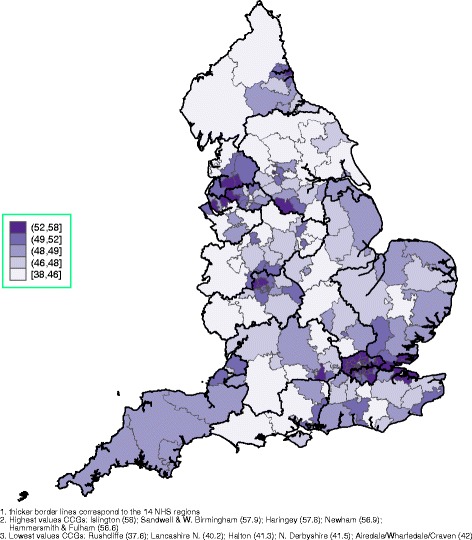
Spatial map at the CCG level, September 2016: mean age of GPs (FTE weighted), EEA or elsewhere qualified^1,^ ^2,^ ^3^

**Fig. 3:**
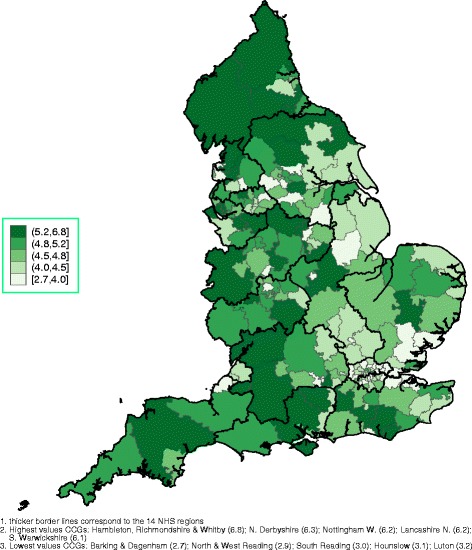
Spatial map at the CCG level, September 2016: cumulative FTE of all GPs per 10,000 patients^1,^ ^2,^ ^3^

**Fig. 4:**
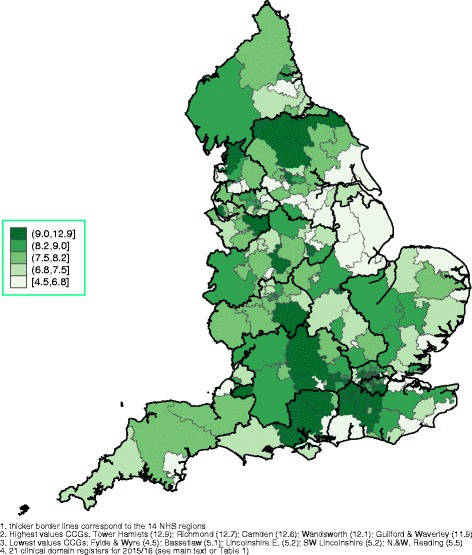
Spatial map at the CCG level, September 2016: cumulative FTE of all GPs per 10,000 counts on the cumulative QOF register^1,^ ^2,^ ^3,^ ^4^

Spatial graphs at the CCG level, with thicker border lines for NHS regions, are provided in the main paper and in Additional file 1. Figure 1 demonstrates the overall FTE of EEA and elsewhere qualified GPs, identifying most of the Greater London area, many CCGs in the East of England and some in the North West and the North East as heavily dependent on non-UK qualified GPs. The mean age of EEA and elsewhere qualified GPs is presented in Fig. 2, with London, areas around London and the North West employing the oldest GPs, on average. The rate of the FTE of all GPs relative to health need (with higher values indicating more GPs) is presented in Fig. 3 (per 10,000 patients) and in Fig. 4 (per 10,000 counts on the cumulative QOF register). The top and bottom 10 CCGs, in terms of dependence on EEA and elsewhere qualified GPs, are provided in Table 3. Additional spatial maps by GP and country of qualification and a complete CCG ranking table on percentage of EEA and elsewhere qualified GPs (Table S3) are provided in Additional file 1.

**Table 3:** Percentage of EEA and elsewhere qualified GPs at the Clinical Commissioning Group level, top and bottom 10

NHS England region	NHS CCG^a^ name	Count of all patients	Cumulative FTE of GPs	FTE % of EEA or else qualified	Mean age, all GPs (FTE weighted)	Mean age, EEA or else qualified GPs (FTE weighted)	Count of GPs aged 55 or over (FTE weighted)	% of EEA or else qualified GPs in those aged 55 or over
Top 10 CCGs in terms of EEA and elsewhere qualified GP recruitment								
Midlands and East (East)	Thurrock	172,877	59.1	*66.5*	51.3	52.8	22.3	71.6
South (South East)	Medway	296,380	107.3	*63.1*	49.9	53.2	36.6	76.0
London	Barking and Dagenham	218,410	58.7	*59.2*	50.0	53.1	21.5	71.8
Midlands and East (North Midlands)	Cannock Chase	132,047	52.3	*56.1*	47.9	50.0	11.6	66.4
North (Yorkshire and Humber)	North Lincolnshire	172,373	83.9	*55.6*	47.1	48.2	21.7	41.2
North (Yorkshire and Humber)	North East Lincolnshire	168,984	62.9	*55.1*	47.7	49.4	15.3	72.6
Midlands and East (East)	Southend	186,472	85.8	*52.9*	50.4	52.4	33.0	55.0
South (South Central)	South Reading	142,173	42.3	*52.7*	48.0	49.2	10.4	65.9
South (South East)	Swale	111,294	41.9	*52.4*	49.8	55.4	11.8	90.6
South (South East)	Thanet	143,947	69.6	*52.3*	49.2	48.0	20.4	41.4
Bottom 10 CCGs in terms of EEA and elsewhere qualified GP recruitment								
North (Cheshire and Merseyside)	Eastern Cheshire	207,052	114.7	*6.0*	44.0	44.0	13.9	0.0
North (Yorkshire and Humber)	Airedale, Wharfedale and Craven	158,536	82.4	*5.9*	43.1	42.0	10.3	4.3
North (Yorkshire and Humber)	Hambleton, Richmondshire and Whitby	143,673	97.5	*5.6*	42.2	44.8	15.7	0.0
North (Yorkshire and Humber)	Harrogate and Rural District	161,837	95.2	*4.8*	41.9	42.3	9.3	0.0
North (Greater Manchester)	South Manchester	174,673	75.2	*4.5*	44.7	46.2	24.3	5.7
South (Wessex)	West Hampshire	556,435	316.5	*4.3*	42.9	43.6	52.1	3.6
South (South Central)	North and West Reading	110,362	32.4	*3.9*	47.0	48.0	5.5	0.0
North (Cumbria and North East)	Northumberland	323,273	195.5	*3.2*	44.8	44.5	30.2	0.0
South (Wessex)	South Eastern Hampshire	213,394	93.1	*2.5*	44.8	50.5	18.6	8.2
North (Yorkshire and Humber)	Vale of York	348,737	156.9	*2.5*	44.1	45.4	21.9	0.0

^a^Clinical Commissioning Group, a middle level NHS organisation

Data in italics is the column of primary interest

GP area of qualification was unknown for 1202 GPs (3.18%), although this has little or no bearing on our analyses (see limitations). The CCG location was unknown for 221 GPs (0.58%), which were necessarily excluded from the spatial maps. Estimates at the practice level could only be computed for a subgroup of 6477 practices (86.1% of all 7527 practices) for which all information was available: workforce with non-missing GP numbers (which is the biggest problem with 1034 practices removed because GP information is missing), patient location deprivation, QOF and payments.

## Discussion

Our results show that in areas that have a higher proportion of EEA and elsewhere qualified GPs, they tend to be older and more of them work full time. These areas have populations that are more deprived, with GPs dealing with deprived patients with poorer health. These areas also have fewer GPs relative to the health needs of the population (as quantified by the QOF). The data also suggest that these doctors tend to have fewer resources and are paid on average less than their UK qualified counterparts. The areas that are most affected by these demographics and workforce characteristics, because they are heavily dependent on non-UK qualified GPs, are concentrated in most of the Greater London area, many CCGs in the East of England and some in the North West and the North East.

### Strengths and limitations of the study

The main strength of this study is the use of numerous national administrative datasets of high data quality that allows us to obtain a complete picture for the whole of England. Nevertheless, some limitations exist.

First, data were not available for all GPs, the headcount of which was 41,865 on 30 September 2016 [12]. Information was reported for 37,792 GPs or 90.3% of all those practicing in England, for 1202 (3.2%) of which the country of qualification was unknown. However, we think missing data are unlikely to introduce bias to our findings; for example, it seems unlikely that most of the GPs for which we could not obtain data were UK qualified and working in deprived areas.

Second, as with all analyses of observational data, we report associations and not causal paths. However, the directionality can be reasonably assumed in most cases (e.g. non-UK qualified GPs do not lead to an increase in deprivation).

Third, aggregating the observed practice-level association to the CCG level to make them more relevant to the current organisational structure of the NHS is challenging. This is due to the fact that CCGs are higher level units which usually serve highly heterogeneous areas in terms of deprivation and populations. Therefore, associations between country of qualification and patients’ health needs or deprivation can be obscured at the CCG level.

### Findings

The over-dependence on overseas qualified GPs has the potential to have an impact on and exacerbate the current recruitment and retention crises in general practice, especially in the short term (the next 10 years). Unfortunately, it is likely to affect areas of the country which have the greatest health needs, thus adding another dimension to Julian Tudor Hart’s inverse care law [22, 23].

General practice remains critical to the long-term sustainability of the NHS [24]. Despite longstanding initiatives to increase GP training numbers to 3250 per annum, GP recruitment has remained well below this target at around 2700 per annum [25]. The crisis in GP recruitment prompted the Secretary of State for Health to promise to increase the number of GPs by 5000 by 2020, in the run-up to the 2015 elections [26]. However, current evidence suggests that the GP workforce did not increase between September 2015 and September 2016, but even fell by 0.3% (FTE) [27]. The question is, how will the 2020 target be met, especially in the context of hardening public attitudes to immigration? Even though the Secretary of State has promised 1500 new medical graduates per year from 2018 [28], it will take at least 10 years for this cohort to be trained in general practice, assuming that we can persuade more than the current 30% of UK graduates to choose general practice as their first career choice [29–31].

So, at least in the short term, the demand for EEA and non-EEA international graduates is likely to increase substantially. Furthermore, the adverse political climate around immigration and Brexit is already resulting in a large number of EU citizens leaving the NHS — recent figures suggest that there has been an 83% increase in doctors from the EU leaving NHS England [27]. This will have an additional impact on retention of the medical workforce, potentially exacerbating problems in shortage areas, especially considering that 36% of GPs aged 50–54 and 80% aged 55 or older intend to retire from general practice in the next five years [10]. As demonstrated by our findings, a disproportionate number of these older GPs are EEA and elsewhere qualified. In a specialty that has always relied on medical migration to cover the shortfall in the GP medical workforce, immigration policy changes that we have described earlier, together with our findings, have the potential to exacerbate the recruitment and retention problems in general practice. Worryingly, as far as we can tell, these important issues have not registered in the calculations of the Department of Health or with HEE.

Our data also suggest a continuing hypocrisy in the way that non-UK qualified doctors are treated. We are dependent on overseas qualified doctors because policy makers are unwilling to take measures to promote self-sufficiency in the medical workforce. The reasons are very likely financial, with one estimate suggesting that non-UK qualified doctors may have contributed £15 billion in saved medical school fees [32]. Yet despite the positive financial contribution they make, arguably even higher than the positive contribution of the average EEA migrant [33], and the fact that they work in deprived areas with patients who have greater needs (and are probably paid less for doing this), they remain a marginalised and stigmatised group of doctors [34]. Despite the recruitment crises in general practice, they have a higher failure rate in the postgraduate exams for general practice, even when adjusted for academic ability. They are more likely to be disciplined by their employers and be brought before the GMC [35]. The reality is that overseas qualified doctors are part of the solution to the recruitment crises facing general practice, and this needs to be acknowledged by policy makers and our politicians.

## Conclusions

This study adds insight into the role of non-UK qualified doctors in delivering care in English general practices, particularly in deprived areas. One in five GPs qualified outside the UK; non-UK qualified GPs work more often on a full-time basis, with more deprived populations and are paid less in comparison to UK qualified GPs. Challenges in directing medical trainees to general practice and also retaining qualified doctors are major drivers of the present workforce crisis in primary care. The current political climate can only worsen this problem, since the retention and the recruitment of non-UK qualified doctors is likely to be affected. A strong association between deprivation and the Brexit vote has been widely reported [36], and our findings point towards a Brexit ’paradox’; the people residing in deprived areas, who mainly voted for Brexit, appear to be more dependent on non-UK qualified doctors to cover their health care needs. The most alarming implication is that there appears no realistic way to increase the number of UK qualified GPs for at least a decade, while the retention and replacement crisis of non-UK qualified GPs is likely to be politically exacerbated in the near future, with patients in more deprived areas more likely to be affected. Non-UK qualified doctors have been a valuable remedy to the shortage of GPs in England, and this remedy is now under threat.

## Additional file

Additional file 1: Additional tables and spatial graphs. (DOCX 3450 kb)

## Abbreviations

A&E: Accident and emergency
CCG: Clinical Commissioning Group
EEA: European Economic Area
EU: European Union
FTE: Full time equivalent
GDP: Gross domestic product
GMC: General Medical Council
GP: General practitioner
HEE: Health Education England
IMG: International Medical Graduates
IMD: Index of Multiple Deprivation
LSOA: Lower Super Output Area
NHS: National Health Service
ONS: Office of National Statistics
PLAB: Professional and Linguistic Assessment Board
QOF: Quality and Outcomes Framework
UK: United Kingdom
