# Supplementary material for: The potential impact of Brexit and immigration policies on the GP workforce in England: a cross-sectional observational study of GP qualification region and the characteristics of the areas and population they served in September 2016
Source: BMC Med. 2017 Nov 16;15:191. doi: 10.1186/s12916-017-0953-y (PMC5688716; doi:10.1186/s12916-017-0953-y)

# Online Appendix for “The potential impact of Brexit and Immigration policies on the GP workforce in England: a cross sectional observational study of GP qualification region and the characteristics of the areas and population they served in September 2016”

Table A1: Individual and practice-level characteristics by NHS region (part 1/2)*

| **Variable name** | **Qualification** | | **Q70: Wessex** | **Q71: London** | **Q72: Yorkshire & Humber** | **Q74: Cumbria & North-East** | **Q75: Cheshire & Merseyside** | **Q76: North Midlands** | **Q77: West Midlands** |
| --- | --- | --- | --- | --- | --- | --- | --- | --- | --- |
| *Individual-level aggregates* | |  |  |  |  |  |  |  |  |
| Number of GPs  (percentage) | All | | 1706  (100.0%) | 6342  (100.0%) | 3506  (100.0%) | 2158  (100.0%) | 2259  (100.0%) | 2300  (100.0%) | 2692  (100.0%) |
|  | UK | | 1496  (87.7%) | 4755  (75.0%) | 2724  (77.7%) | 1691  (78.4%) | 1655  (73.3%) | 1691  (73.5%) | 1943  (72.2%) |
|  | EEA | | 51  (3.0%) | 264  (4.2%) | 134  (3.8%) | 95  (4.4%) | 101  (4.5%) | 93  (4.0%) | 74  (2.7%) |
|  | Elsewhere | | 118  (6.9%) | 1166  (18.4%) | 559  (15.9%) | 325  (15.1%) | 397  (17.6%) | 456  (19.8%) | 584  (21.7%) |
|  | unknown | | 41  (2.4%) | 157  (2.5%) | 89  (2.5%) | 47  (2.2%) | 106  (4.7%) | 60  (2.6%) | 91  (3.4%) |
| Percentage male GPs | All | | 42 | 39.5 | 48.3 | 46.1 | 44.1 | 48.8 | 49.5 |
|  | UK | | 41.6 | 35.5 | 45.3 | 43 | 42.2 | 46.4 | 48.4 |
|  | EEA | | 43.1 | 50.4 | 59 | 63.2 | 54.5 | 49.5 | 51.4 |
|  | Elsewhere | | 49.2 | 53.1 | 62.6 | 59.1 | 48.9 | 58.6 | 54.8 |
|  | unknown | | 36.6 | 40.8 | 36.4 | 34 | 46.7 | 41.7 | 37.4 |
| Median GP age  (25th, 75th centiles) | All | | 44  (37,52) | 40  (32,52) | 43  (36,52) | 45  (38,53) | 40  (33,50) | 44  (37,52) | 44  (36,53) |
|  | UK | | 44  (36,52) | 36  (31,47) | 43  (35,52) | 45  (37,53) | 39  (32,50) | 45  (36,53) | 42  (35,52) |
|  | EEA | | 48  (41,54) | 50  (43,56) | 50  (44,54) | 49  (43,56) | 43  (34,54) | 48  (41,54) | 50  (42,54) |
|  | Elsewhere | | 46  (40,51) | 52  (44,65) | 44  (40,54) | 45  (40,54) | 42  (37,51) | 44  (39,51) | 48  (40,63) |
|  | unknown | | 32  (30,39) | 39  (32,51) | 42  (34,54) | 40  (33,45) | 39  (33,47) | 37  (30,49) | 38  (32,50) |
| Median GP FTE  (25th, 75th centiles) | All | | 0.80  (0.64,1.00) | 0.85  (0.55,1.07) | 0.78  (0.60,1.00) | 0.80  (0.64,1.00) | 0.93  (0.64,1.07) | 0.85  (0.64,1.07) | 0.80  (0.55,1.00) |
|  | UK | | 0.80  (0.64,1.00) | 0.85  (0.56,1.07) | 0.78  (0.56,0.99) | 0.80  (0.64,1.00) | 0.96  (0.64,1.07) | 0.85  (0.64,1.07) | 0.80  (0.56,1.00) |
|  | EEA | | 0.76  (0.59,1.07) | 0.87  (0.53,1.01) | 0.89  (0.67,1.00) | 0.99  (0.67,1.00) | 0.99  (0.64,1.07) | 0.93  (0.72,1.07) | 0.88  (0.56,1.07) |
|  | Elsewhere | | 0.91  (0.67,1.07) | 0.88  (0.53,1.00) | 0.89  (0.64,1.00) | 0.99  (0.67,1.00) | 0.96  (0.64,1.07) | 0.97  (0.64,1.07) | 0.84  (0.53,1.00) |
|  | unknown | | 0.93  (0.64,1.00) | 0.55  (0.40,0.93) | 0.72  (0.43,0.99) | 0.75  (0.60,0.99) | 0.80  (0.53,1.00) | 0.89  (0.64,1.00) | 0.75  (0.50,0.99) |
| *Per 10,000 patients* | |  |  |  |  |  |  |  |  |
| Median number of GPs  (25th,75th centiles) | All | | 7.0  (5.8,8.5) | 6.6  (5.1,8.4) | 6.9  (5.2,8.6) | 7.0  (5.3,8.9) | 7.1  (5.6,8.9) | 6.5  (5.0,8.4) | 6.8  (5.2,8.9) |
| Median FTE of GPs  (25th,75th centiles) | All | | 5.8  (4.6,7.0) | 4.9  (3.8,6.0) | 5.2  (4.1,6.3) | 5.6  (4.4,6.8) | 5.7  (4.5,6.9) | 5.4  (4.2,6.7) | 5.4  (4.3,6.8) |
| *Per 1000 patients aged 75 or older* | |  |  |  |  |  |  |  |  |
| Median number of GPs  (25th,75th centiles) | All | | 7.6  (5.7,10.1) | 13.8  (9.1,21.1) | 8.8  (6.5,12.2) | 7.9  (5.9,10.1) | 8.7  (6.1,12.3) | 7.6  (5.8,10.2) | 8.9  (6.4,13.9) |
| Median FTE of GPs  (25th,75th centiles) | All | | 6.2  (4.6,8.1) | 9.9  (7.0,15.2) | 6.6  (5.1,8.9) | 6.2  (5.0,8.1) | 7.0  (5.1,9.4) | 6.2  (4.8,8.4) | 7.3  (5.4,10.2) |
| *Per 10,000 counts on the cumulative QOF register*† | |  |  |  |  |  |  |  |  |
| Median number of GPs  (25th,75th centiles) | All | | 12.0  (9.4,14.8) | 14.9  (11.1,19.9) | 10.8  (8.3,14.1) | 9.8  (7.7,12.7) | 10.7  (8.1,13.6) | 10.3  (7.8,13.2) | 11.2  (8.5,15.3) |
| Median FTE of GPs  (25th,75th centiles) | All | | 9.6  (7.8,12.1) | 10.8  (8.2,14.2) | 8.2  (6.6,10.3) | 8.0  (6.4,10.0) | 8.7  (6.6,10.5) | 8.3  (6.5,10.8) | 8.7  (6.9,11.3) |
| *Residence overall deprivation of the average patient*‡§ | |  |  |  |  |  |  |  |  |
| Median  (25th, 75th centiles) | All | | 14.4  (9.8,22.7) | 23.9  (15.8,31.0) | 24.3  (15.3,33.5) | 26.2  (18.8,32.1) | 26.1  (15.0,36.7) | 19.6  (14.3,26.8) | 25.2  (16.4,36.8) |
|  | UK | | 14.0  (9.5,22.3) | 22.8  (15.2,30.2) | 21.8  (14.7,32.0) | 25.2  (16.7,31.5) | 23.1  (13.5,33.3) | 18.8  (13.6,24.3) | 22.4  (15.4,34.8) |
|  | EEA | | 18.6  (13.7,23.7) | 26.1  (18.8,32.8) | 28.7  (19.7,36.6) | 27.5  (23.3,31.4) | 34.3  (23.9,38.2) | 22.3  (14.8,29.3) | 29.7  (18.5,37.8) |
|  | Elsewhere | | 18.2  (13.6,25.5) | 25.5  (18.5,32.3) | 30.9  (23.6,39.3) | 30.0  (25.2,36.3) | 35.9  (21.2,47.5) | 23.9  (17.6,32.9) | 34.5  (23.7,40.9) |
|  | unknown | | 13.4  (8.2,15.6) | 25.5  (16.3,33.7) | 27.9  (14.9,34.8) | 25.9  (16.2,32.9) | 36.8  (19.4,53.2) | 19.4  (13.8,25.1) | 23.1  (12.6,37.5) |
| *Average pay per patient*‡¶ | |  |  |  |  |  |  |  |  |
| Median  (25th, 75th centiles) | All | | 133  (122,151) | 129  (117,143) | 148  (130,167) | 134  (124,149) | 133  (122,151) | 138  (126,160) | 135  (121,154) |
|  | UK | | 132  (122,151) | 130  (118,144) | 149  (130,169) | 134  (125,148) | 131  (122,147) | 139  (129,162) | 136  (122,156) |
|  | EEA | | 139  (128,155) | 131  (120,146) | 145  (128,166) | 136  (128,148) | 142  (122,159) | 134  (120,152) | 134  (119,142) |
|  | Elsewhere | | 127  (118,142) | 126  (114,139) | 145  (128,164) | 134  (122,150) | 143  (128,158) | 133  (121,152) | 132  (119,150) |
|  | unknown | | 141  (132,154) | 130  (118,138) | 148  (135,172) | 128  (115,142) | 145  (130,165) | 135  (128,152) | 134  (127,152) |

* Aggregates for England are provided in the main paper

† 21 QOF registers for 2015/16

‡ weighted on: list size for all qualified GPs; the product of list size and the percentage of the respective GP group within the practice (on number rather than FTE), for UK, EEA and elsewhere qualified

§ Index of Multiple Deprivation, details available in the 2015 technical report of the English Indices of Deprivation [^3^](#_ENREF_3)

¶ Minus prescription and dispension costs

Table A2: Individual and practice-level characteristics by NHS region (part 2/2)*

| **Variable name** | **Qualification** | | **Q78: Central Midlands** | **Q79: East** | **Q80: South West** | **Q81: South East** | **Q82: South Central** | **Q83: Greater Manchester** | **Q84: Lancashire** |
| --- | --- | --- | --- | --- | --- | --- | --- | --- | --- |
| *Individual-level aggregates* | |  |  |  |  |  |  |  |  |
| Number of GPs  (percentage) | All | | 2543  (100.0%) | 3266  (100.0%) | 2082  (100.0%) | 2929  (100.0%) | 2672  (100.0%) | 2208  (100.0%) | 908  (100.0%) |
|  | UK | | 1823  (71.7%) | 2208  (67.6%) | 1857  (89.2%) | 2225  (76.0%) | 2335  (87.4%) | 1506  (68.2%) | 576  (63.4%) |
|  | EEA | | 102  (4.0%) | 223  (6.8%) | 84  (4.0%) | 131  (4.5%) | 83  (3.1%) | 67  (3.0%) | 24  (2.6%) |
|  | Elsewhere | | 537  (21.1%) | 752  (23.0%) | 74  (3.6%) | 507  (17.3%) | 224  (8.4%) | 445  (20.2%) | 215  (23.7%) |
|  | unknown | | 81  (3.2%) | 83  (2.5%) | 67  (3.2%) | 66  (2.3%) | 30  (1.1%) | 190  (8.6%) | 93  (10.2%) |
| Percentage male GPs | All | | 52.4 | 45.7 | 45.8 | 46.2 | 39.1 | 49.1 | 56.9 |
|  | UK | | 50 | 44.9 | 45.6 | 44.3 | 38.2 | 47.9 | 54.7 |
|  | EEA | | 49 | 47.1 | 42.9 | 48.1 | 42.2 | 46.3 | 50 |
|  | Elsewhere | | 60.7 | 48.4 | 62.2 | 53.3 | 46.9 | 54.2 | 67.4 |
|  | unknown | | 55.6 | 38.6 | 37.3 | 51.5 | 43.3 | 47.4 | 48.4 |
| Median GP age  (25th, 75th centiles) | All | | 45  (38,53) | 42  (34,52) | 45  (38,53) | 45  (38,53) | 43  (34,52) | 40  (34,50) | 45  (37,53) |
|  | UK | | 45  (37,54) | 41  (32,52) | 45  (38,53) | 44  (37,53) | 43  (34,52) | 38  (32,49) | 44  (36,53) |
|  | EEA | | 48  (41,53) | 45  (36,52) | 49  (43,54) | 49  (43,54) | 46  (38,51) | 44  (39,52) | 48  (42,52) |
|  | Elsewhere | | 44  (40,51) | 43  (37,51) | 47  (41,54) | 47  (41,56) | 46  (41,54) | 43  (38,53) | 46  (39,56) |
|  | unknown | | 39  (32,48) | 41  (34,50) | 36  (32,43) | 42  (37,50) | 39  (31,46) | 44  (35,51) | 48  (41,55) |
| Median GP FTE  (25th, 75th centiles) | All | | 0.88  (0.64,1.00) | 0.99  (0.64,1.07) | 0.80  (0.53,1.00) | 0.77  (0.56,1.00) | 0.80  (0.60,1.00) | 0.85  (0.64,1.07) | 0.87  (0.61,1.01) |
|  | UK | | 0.85  (0.64,1.00) | 0.99  (0.64,1.07) | 0.80  (0.53,0.99) | 0.75  (0.56,0.99) | 0.80  (0.60,1.01) | 0.85  (0.64,1.07) | 0.84  (0.60,1.00) |
|  | EEA | | 0.99  (0.67,1.07) | 1.00  (0.69,1.07) | 0.80  (0.64,0.98) | 0.78  (0.61,1.00) | 0.84  (0.64,1.00) | 0.85  (0.64,1.07) | 0.91  (0.53,1.00) |
|  | Elsewhere | | 0.93  (0.67,1.00) | 1.00  (0.75,1.07) | 0.90  (0.57,1.07) | 0.85  (0.63,1.00) | 0.80  (0.55,1.00) | 0.85  (0.60,1.07) | 0.99  (0.60,1.07) |
|  | unknown | | 0.80  (0.50,1.00) | 0.75  (0.53,1.00) | 0.75  (0.50,1.00) | 0.67  (0.49,0.99) | 0.80  (0.64,0.99) | 0.83  (0.61,1.00) | 1.00  (0.64,1.07) |
| *Per 10,000 patients* | |  |  |  |  |  |  |  |  |
| Median number of GPs  (25th,75th centiles) | All | | 6.1  (4.6,7.5) | 6.2  (4.8,7.8) | 7.8  (6.2,9.2) | 6.5  (5.2,8.0) | 7.3  (5.8,8.9) | 6.3  (4.9,7.9) | 5.7  (4.6,7.2) |
| Median FTE of GPs  (25th,75th centiles) | All | | 4.9  (3.8,5.9) | 5.1  (4.0,6.1) | 5.8  (4.8,6.8) | 4.8  (4.1,5.7) | 5.5  (4.5,6.8) | 4.9  (3.9,6.1) | 5.0  (3.7,6.2) |
| *Per 1000 patients aged 75 or older* | |  |  |  |  |  |  |  |  |
| Median number of GPs  (25th,75th centiles) | All | | 8.1  (6.1,11.0) | 7.1  (5.2,9.5) | 7.6  (6.0,9.9) | 7.2  (5.6,9.7) | 8.8  (6.9,12.0) | 9.6  (6.9,14.9) | 7.4  (5.7,10.9) |
| Median FTE of GPs  (25th,75th centiles) | All | | 6.5  (5.0,8.6) | 5.8  (4.4,7.5) | 5.9  (4.8,7.7) | 5.4  (4.3,7.0) | 7.0  (5.4,9.2) | 7.8  (5.8,11.2) | 6.2  (4.7,9.0) |
| *Per 10,000 counts on the cumulative QOF register*† | |  |  |  |  |  |  |  |  |
| Median number of GPs  (25th,75th centiles) | All | | 10.3  (7.9,13.3) | 10.3  (8.0,13.0) | 11.7  (9.5,14.5) | 11.4  (8.8,14.7) | 13.5  (10.5,16.3) | 10.4  (7.7,13.9) | 8.5  (7.3,11.4) |
| Median FTE of GPs  (25th,75th centiles) | All | | 8.5  (6.6,10.3) | 8.4  (6.8,10.4) | 9.0  (7.5,10.9) | 8.4  (6.7,10.5) | 10.1  (8.2,12.6) | 8.4  (6.3,10.4) | 7.7  (5.6,9.4) |
| *Residence overall deprivation of the average patient*‡§ | |  |  |  |  |  |  |  |  |
| Median  (25th, 75th centiles) | All | | 15.8  (10.6,22.6) | 16.3  (11.9,24.6) | 19.4  (15.1,25.4) | 13.6  (9.8,20.1) | 12.1  (9.1,15.6) | 28.3  (20.4,37.9) | 26.5  (16.2,34.8) |
|  | UK | | 14.4  (9.9,20.0) | 15.5  (11.3,22.1) | 19.3  (15.1,25.1) | 12.5  (9.5,19.1) | 12.0  (9.0,15.3) | 26.8  (18.5,35.9) | 22.1  (15.6,33.2) |
|  | EEA | | 17.1  (11.6,24.9) | 19.2  (13.0,25.9) | 21.0  (15.4,26.8) | 15.4  (11.8,22.6) | 12.6  (9.4,15.9) | 29.2  (21.6,36.0) | 34.8  (22.0,38.4) |
|  | Elsewhere | | 20.1  (13.2,26.8) | 19.7  (12.9,28.5) | 20.9  (15.6,28.5) | 19.0  (13.1,24.3) | 14.7  (10.5,19.5) | 32.8  (24.4,43.2) | 31.9  (21.6,38.4) |
|  | unknown | | 17.3  (10.0,25.9) | 21.0  (13.8,25.4) | 16.6  (9.2,22.4) | 14.4  (9.8,17.9) | 13.7  (9.0,15.5) | 28.5  (21.3,37.5) | 26.0  (16.3,31.3) |
| *Average pay per patient*‡¶ | |  |  |  |  |  |  |  |  |
| Median  (25th, 75th centiles) | All | | 125  (114,144) | 138  (121,170) | 134  (125,152) | 125  (113,143) | 125  (116,139) | 129  (118,139) | 129  (116,143) |
|  | UK | | 125  (114,145) | 145  (124,178) | 135  (125,152) | 126  (115,143) | 126  (117,142) | 130  (117,140) | 133  (118,146) |
|  | EEA | | 124  (113,145) | 134  (122,158) | 134  (125,159) | 125  (114,139) | 126  (115,136) | 126  (122,139) | 130  (114,139) |
|  | Elsewhere | | 123  (113,137) | 126  (113,147) | 130  (119,147) | 122  (111,142) | 120  (109,133) | 128  (118,139) | 125  (114,136) |
|  | unknown | | 135  (114,151) | 124  (112,138) | 133  (124,156) | 123  (111,138) | 127  (117,136) | 125  (120,133) | 127  (116,139) |

* Aggregates for England are provided in the main paper

† 21 QOF registers for 2015/16

‡ weighted on: list size for all qualified GPs; the product of list size and the percentage of the respective GP group within the practice (on number rather than FTE), for UK, EEA and elsewhere qualified

§ Index of Multiple Deprivation, details available in the 2015 technical report of the English Indices of Deprivation [^3^](#_ENREF_3)

¶ Minus prescription and dispension costs

Table A2: Percentage of EEA and Elsewhere qualified GPs at the Clinical Commissioning Group level

| **Rank** | **NHS England region** | **NNH CCG* name** | **Count of all patients** | **Cumulative FTE of GPs** | **FTE % of EEA or else qualified** | **Mean age, all GPs (FTE weighted)** | **Mean age, EEA or else qualified GPs (FTE weighted)** | **Count of GPs aged 55 or over (FTE weighted)** | **% of EEA or else qualified GPs in those aged 55 or over** |
| --- | --- | --- | --- | --- | --- | --- | --- | --- | --- |
| 1 | Midlands and East (East) | Thurrock | 172877 | 59.1 | 66.5 | 51.3 | 52.8 | 22.3 | 71.6 |
| 2 | South (South East) | Medway | 296380 | 107.3 | 63.1 | 49.9 | 53.2 | 36.6 | 76.0 |
| 3 | London | Barking and Dagenham | 218410 | 58.7 | 59.2 | 50.0 | 53.1 | 21.5 | 71.8 |
| 4 | Midlands and East (North Midlands) | Cannock Chase | 132047 | 52.3 | 56.1 | 47.9 | 50.0 | 11.6 | 66.4 |
| 5 | North (Yorkshire and Humber) | North Lincolnshire | 172373 | 83.9 | 55.6 | 47.1 | 48.2 | 21.7 | 41.2 |
| 6 | North (Yorkshire and Humber) | North East Lincolnshire | 168984 | 62.9 | 55.1 | 47.7 | 49.4 | 15.3 | 72.6 |
| 7 | Midlands and East (East) | Southend | 186472 | 85.8 | 52.9 | 50.4 | 52.4 | 33.0 | 55.0 |
| 8 | South (South Central) | South Reading | 142173 | 42.3 | 52.7 | 48.0 | 49.2 | 10.4 | 65.9 |
| 9 | South (South East) | Swale | 111294 | 41.9 | 52.4 | 49.8 | 55.4 | 11.8 | 90.6 |
| 10 | South (South East) | Thanet | 143947 | 69.6 | 52.3 | 49.2 | 48.0 | 20.4 | 41.4 |
| 11 | Midlands and East (Central Midlands) | Milton Keynes | 287844 | 115.4 | 51.8 | 46.4 | 48.3 | 29.0 | 58.3 |
| 12 | Midlands and East (East) | Castle Point and Rochford | 184444 | 94.7 | 51.6 | 47.3 | 53.3 | 30.6 | 79.2 |
| 13 | Midlands and East (North Midlands) | Stoke on Trent | 287532 | 142.7 | 50.8 | 48.3 | 49.6 | 46.1 | 51.1 |
| 14 | Midlands and East (West Midlands) | Walsall | 281150 | 132.7 | 48.9 | 45.3 | 51.2 | 32.1 | 69.4 |
| 15 | London | Havering | 273109 | 108.3 | 48.4 | 49.1 | 55.9 | 36.8 | 64.5 |
| 16 | Midlands and East (Central Midlands) | Lincolnshire East | 246854 | 100.6 | 47.4 | 48.6 | 47.8 | 30.4 | 36.4 |
| 17 | North (Yorkshire and Humber) | Bradford City | 127163 | 45.3 | 46.0 | 48.6 | 53.4 | 11.8 | 67.5 |
| 18 | Midlands and East (East) | Basildon and Brentwood | 276097 | 113.7 | 45.5 | 48.2 | 50.9 | 32.5 | 53.8 |
| 19 | London | Redbridge | 299325 | 113.6 | 44.4 | 47.5 | 50.6 | 34.2 | 48.7 |
| 20 | North (Yorkshire and Humber) | Hull | 294005 | 113.1 | 43.9 | 47.6 | 49.1 | 32.3 | 34.0 |
| 21 | London | Waltham Forest | 305127 | 108.5 | 43.4 | 48.3 | 56.2 | 38.5 | 68.3 |
| 22 | South (South East) | Ashford | 130426 | 55.8 | 43.2 | 46.2 | 46.3 | 13.3 | 44.7 |
| 23 | London | Bexley | 236961 | 84.3 | 42.0 | 45.3 | 49.7 | 19.6 | 53.0 |
| 24 | London | Enfield | 330850 | 137.6 | 41.7 | 47.6 | 55.3 | 45.4 | 65.0 |
| 25 | South (South East) | South Kent Coast | 203385 | 86.8 | 41.5 | 48.7 | 51.8 | 24.0 | 51.7 |
| 26 | North (Yorkshire and Humber) | Doncaster | 316567 | 145.7 | 41.0 | 44.5 | 46.3 | 31.6 | 39.2 |
| 27 | North (Cumbria and North East) | Hartlepool and Stockton-on-Tees | 295439 | 143.8 | 40.8 | 45.8 | 47.2 | 27.0 | 46.2 |
| 28 | Midlands and East (Central Midlands) | Luton | 229824 | 73.7 | 40.7 | 47.6 | 51.0 | 19.5 | 49.9 |
| 29 | London | Newham | 379615 | 125.4 | 40.0 | 47.7 | 56.9 | 37.5 | 69.9 |
| 30 | South (South East) | Crawley | 130929 | 55.6 | 39.3 | 46.8 | 46.9 | 9.6 | 30.7 |
| 31 | North (Yorkshire and Humber) | North Kirklees | 191546 | 68.7 | 38.5 | 46.7 | 52.3 | 18.1 | 62.4 |
| 32 | North (Lancashire) | Blackburn with Darwen | 172974 | 65.9 | 38.4 | 48.5 | 53.1 | 19.0 | 54.9 |
| 33 | South (Wessex) | Isle of Wight | 143240 | 74.7 | 38.2 | 46.1 | 47.0 | 13.4 | 18.1 |
| 34 | London | Greenwich | 287293 | 118.1 | 37.5 | 45.6 | 55.4 | 32.7 | 75.5 |
| 35 | Midlands and East (East) | North East Essex | 343782 | 133.4 | 36.4 | 46.1 | 47.9 | 25.7 | 45.2 |
| 36 | North (Cumbria and North East) | Sunderland | 284115 | 131.1 | 36.3 | 46.0 | 50.6 | 30.3 | 50.6 |
| 37 | North (Lancashire) | Blackpool | 172350 | 82.7 | 36.2 | 44.2 | 43.4 | 8.2 | 25.6 |
| 38 | London | Brent | 364816 | 154.1 | 35.2 | 47.0 | 55.0 | 47.9 | 60.7 |
| 39 | North (Cumbria and North East) | South Tees | 294593 | 153.3 | 35.2 | 46.9 | 47.5 | 37.6 | 28.0 |
| 40 | Midlands and East (West Midlands) | Sandwell and West Birmingham | 574516 | 214.1 | 34.9 | 47.2 | 57.9 | 66.4 | 66.6 |
| 41 | North (Cheshire and Merseyside) | Knowsley | 163192 | 85.7 | 34.7 | 46.1 | 48.5 | 33.2 | 16.3 |
| 42 | North (Greater Manchester) | Wigan Borough | 324758 | 168.2 | 34.7 | 46.7 | 52.3 | 51.8 | 42.6 |
| 43 | Midlands and East (Central Midlands) | Leicester City | 395033 | 148.0 | 34.7 | 45.7 | 48.1 | 31.3 | 32.6 |
| 44 | South (South Central) | Slough | 155928 | 63.6 | 34.6 | 43.5 | 49.2 | 13.4 | 60.9 |
| 45 | Midlands and East (North Midlands) | Hardwick | 103125 | 54.6 | 34.1 | 43.8 | 47.4 | 2.1 | 100.0 |
| 46 | North (Greater Manchester) | North Manchester | 209877 | 94.2 | 33.5 | 43.3 | 43.9 | 14.9 | 22.8 |
| 47 | North (Cumbria and North East) | Durham Dales, Easington and Sedgefield | 290455 | 164.8 | 33.3 | 46.8 | 49.2 | 42.2 | 43.1 |
| 48 | London | Ealing | 424924 | 174.2 | 32.9 | 47.1 | 53.6 | 38.6 | 49.5 |
| 49 | Midlands and East (East) | West Norfolk | 173536 | 88.2 | 32.4 | 48.0 | 47.7 | 20.6 | 24.5 |
| 50 | North (Lancashire) | West Lancashire | 113135 | 56.3 | 32.3 | 47.2 | 50.7 | 13.8 | 42.6 |
| 51 | North (Yorkshire and Humber) | Barnsley | 257326 | 102.6 | 32.1 | 44.3 | 49.5 | 18.2 | 49.7 |
| 52 | Midlands and East (North Midlands) | South East Staffordshire and Seisdon Peninsula | 217480 | 108.3 | 31.9 | 46.9 | 50.6 | 27.5 | 40.2 |
| 53 | Midlands and East (Central Midlands) | Nene | 669681 | 303.2 | 31.7 | 45.6 | 44.7 | 51.1 | 13.5 |
| 54 | Midlands and East (Central Midlands) | Bedfordshire | 469884 | 193.4 | 31.6 | 45.5 | 47.0 | 41.6 | 28.1 |
| 55 | Midlands and East (West Midlands) | Wolverhampton | 273894 | 124.5 | 31.2 | 44.8 | 49.3 | 27.8 | 34.0 |
| 56 | London | Bromley | 344386 | 164.9 | 30.6 | 46.3 | 52.6 | 45.8 | 48.1 |
| 57 | South (South East) | Dartford, Gravesham and Swanley | 250479 | 91.0 | 30.5 | 45.2 | 48.4 | 24.7 | 37.1 |
| 58 | London | Southwark | 319053 | 119.1 | 30.5 | 46.0 | 54.2 | 34.0 | 46.8 |
| 59 | Midlands and East (West Midlands) | Coventry and Rugby | 491071 | 202.4 | 30.4 | 47.1 | 50.5 | 52.9 | 40.3 |
| 60 | London | Harrow | 261127 | 108.2 | 30.3 | 46.9 | 52.5 | 32.5 | 46.0 |
| 61 | Midlands and East (East) | Mid Essex | 386830 | 146.5 | 30.3 | 47.0 | 49.4 | 35.5 | 33.5 |
| 62 | Midlands and East (North Midlands) | East Staffordshire | 139571 | 81.5 | 30.1 | 46.4 | 47.3 | 14.0 | 39.1 |
| 63 | London | Lewisham | 320456 | 128.9 | 29.7 | 45.7 | 54.3 | 39.2 | 52.6 |
| 64 | Midlands and East (East) | Great Yarmouth and Waveney | 233139 | 99.0 | 29.6 | 46.7 | 46.1 | 21.7 | 18.1 |
| 65 | London | Hillingdon | 305315 | 102.8 | 29.5 | 46.3 | 52.5 | 24.7 | 43.0 |
| 66 | North (Yorkshire and Humber) | Bassetlaw | 115462 | 41.3 | 29.2 | 46.1 | 43.2 | 8.7 | 25.7 |
| 67 | London | Sutton | 188136 | 85.9 | 29.2 | 47.7 | 51.6 | 24.2 | 29.0 |
| 68 | London | Croydon | 404603 | 176.8 | 28.9 | 45.4 | 50.2 | 41.5 | 37.0 |
| 69 | Midlands and East (West Midlands) | Warwickshire North | 188458 | 91.2 | 28.9 | 46.7 | 47.2 | 21.6 | 28.4 |
| 70 | North (Lancashire) | East Lancashire | 377308 | 195.1 | 28.8 | 46.4 | 51.9 | 50.1 | 37.8 |
| 71 | Midlands and East (North Midlands) | North Staffordshire | 217327 | 108.9 | 28.6 | 44.6 | 42.0 | 14.7 | 7.8 |
| 72 | London | Hounslow | 304809 | 93.8 | 28.5 | 47.4 | 54.3 | 27.3 | 51.0 |
| 73 | Midlands and East (Central Midlands) | South West Lincolnshire | 132741 | 48.5 | 28.2 | 47.9 | 46.0 | 12.6 | 0.0 |
| 74 | North (Cheshire and Merseyside) | St Helens | 196350 | 101.4 | 27.8 | 48.7 | 52.5 | 36.9 | 34.5 |
| 75 | Midlands and East (East) | Ipswich and East Suffolk | 403025 | 184.4 | 27.6 | 47.7 | 48.2 | 45.2 | 30.0 |
| 76 | Midlands and East (North Midlands) | Mansfield and Ashfield | 191172 | 76.1 | 27.0 | 45.8 | 50.3 | 16.4 | 39.9 |
| 77 | North (Greater Manchester) | Oldham | 250930 | 95.9 | 27.0 | 44.3 | 47.4 | 13.9 | 24.8 |
| 78 | Midlands and East (West Midlands) | Birmingham South and Central | 309075 | 149.2 | 26.6 | 45.4 | 51.7 | 34.6 | 46.2 |
| 79 | North (Yorkshire and Humber) | Calderdale | 219234 | 87.3 | 26.2 | 44.4 | 47.3 | 15.4 | 37.2 |
| 80 | Midlands and East (North Midlands) | Stafford and Surrounds | 147412 | 70.9 | 25.8 | 46.5 | 45.7 | 13.8 | 14.5 |
| 81 | North (Greater Manchester) | Salford | 265537 | 134.1 | 25.7 | 44.6 | 51.3 | 24.8 | 45.0 |
| 82 | Midlands and East (East) | Norwich | 217442 | 93.0 | 25.6 | 47.1 | 48.5 | 23.6 | 26.4 |
| 83 | North (Greater Manchester) | Tameside and Glossop | 245511 | 112.9 | 25.6 | 45.8 | 49.1 | 22.5 | 46.0 |
| 84 | Midlands and East (Central Midlands) | Corby | 75918 | 43.6 | 25.1 | 43.1 | 45.4 | 6.6 | 19.3 |
| 85 | North (Lancashire) | Greater Preston | 212470 | 114.9 | 24.9 | 46.1 | 49.7 | 40.6 | 27.0 |
| 86 | South (South East) | Hastings and Rother | 161016 | 80.4 | 24.9 | 46.4 | 49.3 | 23.3 | 24.0 |
| 87 | London | Hammersmith and Fulham | 209277 | 82.1 | 24.8 | 44.7 | 56.6 | 17.3 | 53.9 |
| 88 | North (Greater Manchester) | Heywood, Middleton and Rochdale | 229555 | 116.7 | 24.8 | 45.9 | 49.8 | 31.6 | 23.5 |
| 89 | London | Kingston | 203150 | 75.4 | 24.6 | 44.5 | 54.4 | 14.2 | 59.8 |
| 90 | North (Cheshire and Merseyside) | Liverpool | 517863 | 309.4 | 24.5 | 44.3 | 52.8 | 70.5 | 47.5 |
| 91 | Midlands and East (North Midlands) | Nottingham City | 369242 | 172.7 | 24.3 | 43.7 | 49.6 | 30.0 | 41.1 |
| 92 | South (South East) | Canterbury and Coastal | 221574 | 114.3 | 24.0 | 46.4 | 45.8 | 27.8 | 15.8 |
| 93 | London | Haringey | 310331 | 118.6 | 23.9 | 45.2 | 57.8 | 29.8 | 56.2 |
| 94 | Midlands and East (West Midlands) | Birmingham CrossCity | 717106 | 353.3 | 23.8 | 45.8 | 51.9 | 75.5 | 35.6 |
| 95 | North (Yorkshire and Humber) | Rotherham | 260511 | 121.9 | 23.8 | 45.7 | 50.5 | 30.6 | 29.9 |
| 96 | London | Tower Hamlets | 305959 | 151.0 | 23.8 | 43.8 | 51.3 | 25.2 | 53.5 |
| 97 | Midlands and East (East) | West Essex | 307091 | 140.1 | 23.7 | 43.9 | 50.1 | 26.3 | 35.8 |
| 98 | Midlands and East (East) | Cambridgeshire and Peterborough | 940112 | 427.2 | 23.7 | 45.9 | 46.6 | 86.9 | 19.0 |
| 99 | North (Cumbria and North East) | Darlington | 107530 | 59.4 | 23.2 | 47.4 | 48.0 | 13.0 | 28.3 |
| 100 | Midlands and East (West Midlands) | Dudley | 317319 | 161.9 | 23.2 | 44.4 | 52.6 | 37.0 | 48.3 |
| 101 | North (Yorkshire and Humber) | Wakefield | 367370 | 197.3 | 23.1 | 43.0 | 46.8 | 28.6 | 37.2 |
| 102 | South (South East) | East Surrey | 181112 | 93.6 | 23.0 | 44.8 | 43.9 | 17.8 | 8.0 |
| 103 | North (Lancashire) | Fylde & Wyre | 151238 | 53.6 | 22.9 | 44.2 | 42.0 | 11.1 | 0.0 |
| 104 | Midlands and East (East) | South Norfolk | 236782 | 115.9 | 22.8 | 44.6 | 46.8 | 17.8 | 9.8 |
| 105 | Midlands and East (Central Midlands) | East and North Hertfordshire | 591809 | 255.3 | 22.7 | 46.5 | 48.1 | 62.4 | 24.1 |
| 106 | Midlands and East (Central Midlands) | Lincolnshire West | 235088 | 102.9 | 22.5 | 46.0 | 46.8 | 17.8 | 32.5 |
| 107 | North (Greater Manchester) | Bolton | 304873 | 174.7 | 22.3 | 44.2 | 50.0 | 44.9 | 28.3 |
| 108 | Midlands and East (North Midlands) | Nottingham West | 93552 | 58.1 | 22.0 | 45.3 | 45.6 | 9.6 | 6.9 |
| 109 | North (Cheshire and Merseyside) | Halton | 130346 | 66.2 | 21.9 | 44.9 | 41.3 | 17.0 | 8.6 |
| 110 | North (Cheshire and Merseyside) | Warrington | 215863 | 98.7 | 21.5 | 43.8 | 45.0 | 18.3 | 10.9 |
| 111 | North (Yorkshire and Humber) | East Riding of Yorkshire | 301790 | 136.1 | 21.5 | 44.9 | 43.7 | 26.6 | 13.2 |
| 112 | Midlands and East (North Midlands) | Southern Derbyshire | 547961 | 269.2 | 21.3 | 44.6 | 46.6 | 44.1 | 22.7 |
| 113 | Midlands and East (North Midlands) | Telford and Wrekin | 181912 | 86.0 | 21.2 | 44.9 | 44.6 | 11.4 | 27.6 |
| 114 | North (Yorkshire and Humber) | Scarborough and Ryedale | 119206 | 72.0 | 21.1 | 45.7 | 46.1 | 12.8 | 13.9 |
| 115 | North (Greater Manchester) | Bury | 201595 | 97.4 | 21.0 | 44.6 | 47.4 | 32.8 | 16.9 |
| 116 | London | West London | 240733 | 96.1 | 20.8 | 45.9 | 54.0 | 25.8 | 39.4 |
| 117 | Midlands and East (West Midlands) | Solihull | 246810 | 114.0 | 20.8 | 45.0 | 48.2 | 19.9 | 28.7 |
| 118 | North (Cheshire and Merseyside) | South Sefton | 154862 | 71.1 | 20.1 | 47.1 | 48.6 | 16.9 | 28.5 |
| 119 | Midlands and East (North Midlands) | Newark & Sherwood | 132618 | 61.1 | 19.9 | 42.7 | 43.5 | 7.7 | 15.6 |
| 120 | London | Wandsworth | 390560 | 165.1 | 19.5 | 44.9 | 55.1 | 34.4 | 48.2 |
| 121 | South (South Central) | Bracknell and Ascot | 140494 | 60.1 | 19.2 | 45.6 | 52.2 | 12.7 | 21.0 |
| 122 | London | Merton | 222999 | 81.7 | 19.1 | 45.6 | 51.1 | 16.7 | 32.5 |
| 123 | North (Cumbria and North East) | Cumbria | 522739 | 315.1 | 18.8 | 46.1 | 45.6 | 67.3 | 16.5 |
| 124 | South (South Central) | Swindon | 230121 | 109.0 | 18.5 | 45.7 | 46.6 | 24.0 | 14.2 |
| 125 | North (Cumbria and North East) | South Tyneside | 156255 | 80.4 | 17.6 | 45.1 | 53.6 | 16.7 | 34.9 |
| 126 | Midlands and East (West Midlands) | Redditch and Bromsgrove | 175902 | 89.4 | 17.4 | 44.7 | 50.4 | 11.2 | 30.2 |
| 127 | North (Lancashire) | Chorley and South Ribble | 180826 | 75.7 | 16.8 | 46.9 | 56.0 | 24.8 | 29.5 |
| 128 | South (South East) | North West Surrey | 368607 | 168.1 | 16.6 | 44.4 | 47.8 | 29.3 | 30.1 |
| 129 | London | Islington | 242505 | 110.1 | 16.6 | 45.6 | 58.0 | 26.7 | 46.4 |
| 130 | South (Wessex) | Portsmouth | 224172 | 105.7 | 16.3 | 46.1 | 48.2 | 25.5 | 20.6 |
| 131 | London | Barnet | 411166 | 162.8 | 16.2 | 46.6 | 53.8 | 43.9 | 31.8 |
| 132 | London | City and Hackney | 307267 | 131.5 | 16.1 | 43.8 | 53.6 | 24.7 | 43.0 |
| 133 | London | Richmond | 213375 | 100.7 | 16.0 | 46.6 | 54.0 | 28.4 | 32.0 |
| 134 | South (South Central) | Chiltern | 340414 | 158.7 | 15.8 | 44.2 | 45.9 | 26.0 | 22.7 |
| 135 | North (Lancashire) | Lancashire North | 158760 | 97.9 | 15.7 | 44.3 | 40.2 | 14.9 | 0.0 |
| 136 | London | Central London (Westminster) | 210532 | 84.6 | 15.5 | 46.0 | 54.5 | 20.0 | 33.5 |
| 137 | Midlands and East (North Midlands) | Nottingham North and East | 150250 | 64.4 | 15.5 | 43.8 | 42.2 | 10.5 | 0.0 |
| 138 | North (Greater Manchester) | Central Manchester | 234756 | 101.3 | 15.4 | 43.8 | 47.7 | 15.3 | 9.6 |
| 139 | Midlands and East (Central Midlands) | South Lincolnshire | 165008 | 78.3 | 15.4 | 47.4 | 46.3 | 16.3 | 0.0 |
| 140 | South (South Central) | Wokingham | 161601 | 89.0 | 15.0 | 45.6 | 52.9 | 21.9 | 23.7 |
| 141 | North (Cheshire and Merseyside) | Southport and Formby | 123966 | 60.8 | 14.9 | 45.0 | 43.9 | 8.4 | 11.1 |
| 142 | South (South East) | Surrey Downs | 305256 | 142.6 | 14.9 | 45.6 | 47.0 | 27.0 | 16.7 |
| 143 | North (Yorkshire and Humber) | Leeds South and East | 276114 | 138.3 | 14.9 | 43.9 | 44.8 | 29.6 | 12.0 |
| 144 | Midlands and East (Central Midlands) | Herts Valleys | 638131 | 286.3 | 14.9 | 45.8 | 45.2 | 60.1 | 4.0 |
| 145 | South (South East) | West Kent | 483975 | 232.0 | 14.5 | 45.5 | 46.7 | 48.1 | 10.4 |
| 146 | South (South East) | Coastal West Sussex | 491292 | 228.9 | 14.5 | 45.1 | 47.8 | 40.9 | 22.4 |
| 147 | Midlands and East (East) | West Suffolk | 247882 | 136.8 | 14.3 | 44.1 | 45.5 | 22.8 | 7.9 |
| 148 | South (South East) | High Weald Lewes Havens | 169527 | 90.4 | 14.2 | 44.6 | 45.4 | 11.4 | 9.7 |
| 149 | South (Wessex) | Fareham and Gosport | 203825 | 98.5 | 14.2 | 45.6 | 50.1 | 18.8 | 22.0 |
| 150 | North (Yorkshire and Humber) | Bradford Districts | 339234 | 160.1 | 14.1 | 42.1 | 45.4 | 15.3 | 19.6 |
| 151 | North (Greater Manchester) | Trafford | 239504 | 100.0 | 13.8 | 46.5 | 50.0 | 26.5 | 15.2 |
| 152 | London | Lambeth | 395631 | 145.8 | 13.6 | 42.8 | 53.4 | 19.7 | 38.5 |
| 153 | South (South East) | Brighton and Hove | 309371 | 110.5 | 13.6 | 46.2 | 50.3 | 16.5 | 11.9 |
| 154 | North (Cheshire and Merseyside) | Wirral | 334790 | 189.6 | 13.1 | 45.5 | 51.1 | 39.4 | 22.5 |
| 155 | Midlands and East (West Midlands) | South Worcestershire | 304738 | 152.3 | 13.1 | 43.1 | 43.9 | 21.3 | 12.9 |
| 156 | North (Cheshire and Merseyside) | South Cheshire | 182110 | 92.0 | 13.1 | 46.4 | 49.5 | 14.8 | 15.5 |
| 157 | South (Wessex) | Southampton | 280061 | 131.7 | 12.7 | 44.0 | 46.2 | 24.8 | 11.0 |
| 158 | Midlands and East (North Midlands) | Shropshire | 306173 | 187.4 | 12.5 | 45.4 | 44.4 | 40.1 | 8.0 |
| 159 | Midlands and East (North Midlands) | Erewash | 97431 | 48.3 | 12.2 | 44.3 | 47.5 | 4.9 | 36.5 |
| 160 | North (Cumbria and North East) | North Durham | 253329 | 113.8 | 12.1 | 44.0 | 46.5 | 17.2 | 14.7 |
| 161 | Midlands and East (North Midlands) | North Derbyshire | 292082 | 185.2 | 11.9 | 44.6 | 41.5 | 31.9 | 9.7 |
| 162 | South (South Central) | Aylesbury Vale | 210932 | 94.5 | 11.9 | 42.5 | 44.9 | 10.7 | 0.0 |
| 163 | Midlands and East (Central Midlands) | West Leicestershire | 383319 | 188.0 | 11.8 | 44.6 | 46.0 | 35.6 | 14.6 |
| 164 | South (South East) | Eastbourne, Hailsham and Seaford | 185853 | 102.2 | 11.8 | 44.4 | 46.0 | 18.1 | 13.6 |
| 165 | Midlands and East (North Midlands) | Rushcliffe | 124635 | 65.3 | 11.8 | 41.7 | 37.6 | 6.3 | 0.0 |
| 166 | North (Cumbria and North East) | Newcastle Gateshead | 507998 | 264.1 | 11.8 | 44.8 | 49.8 | 47.5 | 18.9 |
| 167 | North (Yorkshire and Humber) | Leeds West | 375545 | 188.9 | 11.7 | 43.3 | 50.6 | 29.4 | 21.5 |
| 168 | South (Wessex) | North Hampshire | 223948 | 120.1 | 11.6 | 42.4 | 48.4 | 16.5 | 21.6 |
| 169 | South (South West) | South Gloucestershire | 266304 | 110.3 | 11.2 | 45.1 | 50.7 | 20.1 | 12.1 |
| 170 | South (South Central) | Windsor, Ascot and Maidenhead | 154511 | 71.8 | 11.1 | 42.9 | 47.3 | 12.3 | 7.5 |
| 171 | North (Yorkshire and Humber) | Sheffield | 595849 | 308.8 | 11.1 | 44.5 | 53.0 | 59.3 | 26.7 |
| 172 | North (Yorkshire and Humber) | Greater Huddersfield | 243436 | 112.5 | 10.9 | 44.4 | 46.4 | 18.9 | 6.6 |
| 173 | North (Yorkshire and Humber) | Leeds North | 213855 | 80.8 | 10.7 | 43.1 | 48.7 | 11.9 | 13.9 |
| 174 | North (Cheshire and Merseyside) | Vale Royal | 104676 | 54.7 | 10.6 | 45.6 | 49.2 | 9.2 | 21.7 |
| 175 | Midlands and East (Central Midlands) | East Leicestershire and Rutland | 326621 | 170.7 | 10.5 | 42.0 | 44.1 | 20.3 | 5.3 |
| 176 | South (South Central) | Newbury and District | 118426 | 56.4 | 10.5 | 44.4 | 44.7 | 9.4 | 5.3 |
| 177 | South (South East) | Horsham and Mid Sussex | 237374 | 129.3 | 10.5 | 44.9 | 50.1 | 26.6 | 10.7 |
| 178 | South (South East) | Surrey Heath | 96249 | 50.2 | 10.3 | 40.6 | 46.8 | 5.2 | 6.4 |
| 179 | Midlands and East (East) | North Norfolk | 172420 | 70.0 | 10.3 | 46.1 | 47.5 | 12.7 | 7.9 |
| 180 | South (Wessex) | North East Hampshire and Farnham | 225650 | 95.9 | 9.5 | 45.7 | 43.5 | 20.1 | 4.9 |
| 181 | London | Camden | 269574 | 130.7 | 9.4 | 42.2 | 49.1 | 22.7 | 10.2 |
| 182 | North (Greater Manchester) | Stockport | 308754 | 143.1 | 9.2 | 43.3 | 50.4 | 19.9 | 20.1 |
| 183 | South (South West) | Kernow | 566166 | 283.5 | 8.9 | 46.4 | 48.4 | 64.8 | 10.6 |
| 184 | South (South West) | North Somerset | 217625 | 80.3 | 8.8 | 45.3 | 51.0 | 22.8 | 8.6 |
| 185 | North (Cheshire and Merseyside) | West Cheshire | 260350 | 114.5 | 8.6 | 43.8 | 43.0 | 16.9 | 3.1 |
| 186 | South (South East) | Guildford and Waverley | 222741 | 131.1 | 8.5 | 44.3 | 46.5 | 22.8 | 8.6 |
| 187 | South (South West) | Somerset | 567692 | 280.7 | 8.5 | 46.8 | 49.4 | 61.9 | 9.9 |
| 188 | South (South Central) | Wiltshire | 481714 | 264.1 | 8.4 | 44.9 | 45.1 | 49.5 | 2.7 |
| 189 | South (Wessex) | Dorset | 789995 | 382.1 | 8.1 | 45.0 | 44.7 | 65.3 | 4.9 |
| 190 | South (South Central) | Gloucestershire | 633546 | 334.7 | 7.9 | 45.3 | 47.4 | 61.1 | 8.6 |
| 191 | South (South West) | Bristol | 499482 | 217.6 | 7.9 | 44.5 | 45.8 | 43.5 | 5.0 |
| 192 | South (South West) | South Devon and Torbay | 283932 | 135.3 | 7.8 | 46.6 | 49.5 | 23.6 | 8.2 |
| 193 | Midlands and East (West Midlands) | Herefordshire | 185328 | 92.2 | 7.1 | 46.5 | 45.4 | 20.0 | 6.5 |
| 194 | South (South Central) | Oxfordshire | 725823 | 361.6 | 6.8 | 44.4 | 48.0 | 64.8 | 8.0 |
| 195 | Midlands and East (West Midlands) | Wyre Forest | 114947 | 69.2 | 6.7 | 43.0 | 45.9 | 7.7 | 0.0 |
| 196 | Midlands and East (West Midlands) | South Warwickshire | 280575 | 172.5 | 6.7 | 43.6 | 47.3 | 25.0 | 13.9 |
| 197 | North (Cumbria and North East) | North Tyneside | 217629 | 117.4 | 6.3 | 44.5 | 51.3 | 20.9 | 18.8 |
| 198 | South (South Central) | Bath and North East Somerset | 199742 | 106.8 | 6.0 | 44.6 | 48.2 | 14.6 | 11.2 |
| 199 | South (South West) | Northern, Eastern and Western Devon | 912870 | 479.5 | 6.0 | 45.6 | 48.7 | 92.8 | 8.3 |
| 200 | North (Cheshire and Merseyside) | Eastern Cheshire | 207052 | 114.7 | 6.0 | 44.0 | 44.0 | 13.9 | 0.0 |
| 201 | North (Yorkshire and Humber) | Airedale, Wharfedale and Craven | 158536 | 82.4 | 5.9 | 43.1 | 42.0 | 10.3 | 4.3 |
| 202 | North (Yorkshire and Humber) | Hambleton, Richmondshire and Whitby | 143673 | 97.5 | 5.6 | 42.2 | 44.8 | 15.7 | 0.0 |
| 203 | North (Yorkshire and Humber) | Harrogate and Rural District | 161837 | 95.2 | 4.8 | 41.9 | 42.3 | 9.3 | 0.0 |
| 204 | North (Greater Manchester) | South Manchester | 174673 | 75.2 | 4.5 | 44.7 | 46.2 | 24.3 | 5.7 |
| 205 | South (Wessex) | West Hampshire | 556435 | 316.5 | 4.3 | 42.9 | 43.6 | 52.1 | 3.6 |
| 206 | South (South Central) | North & West Reading | 110362 | 32.4 | 3.9 | 47.0 | 48.0 | 5.5 | 0.0 |
| 207 | North (Cumbria and North East) | Northumberland | 323273 | 195.5 | 3.2 | 44.8 | 44.5 | 30.2 | 0.0 |
| 208 | South (Wessex) | South Eastern Hampshire | 213394 | 93.1 | 2.5 | 44.8 | 50.5 | 18.6 | 8.2 |
| 209 | North (Yorkshire and Humber) | Vale of York | 348737 | 156.9 | 2.5 | 44.1 | 45.4 | 21.9 | 0.0 |

* Clinical Commissioning Group, a middle level NHS organisation


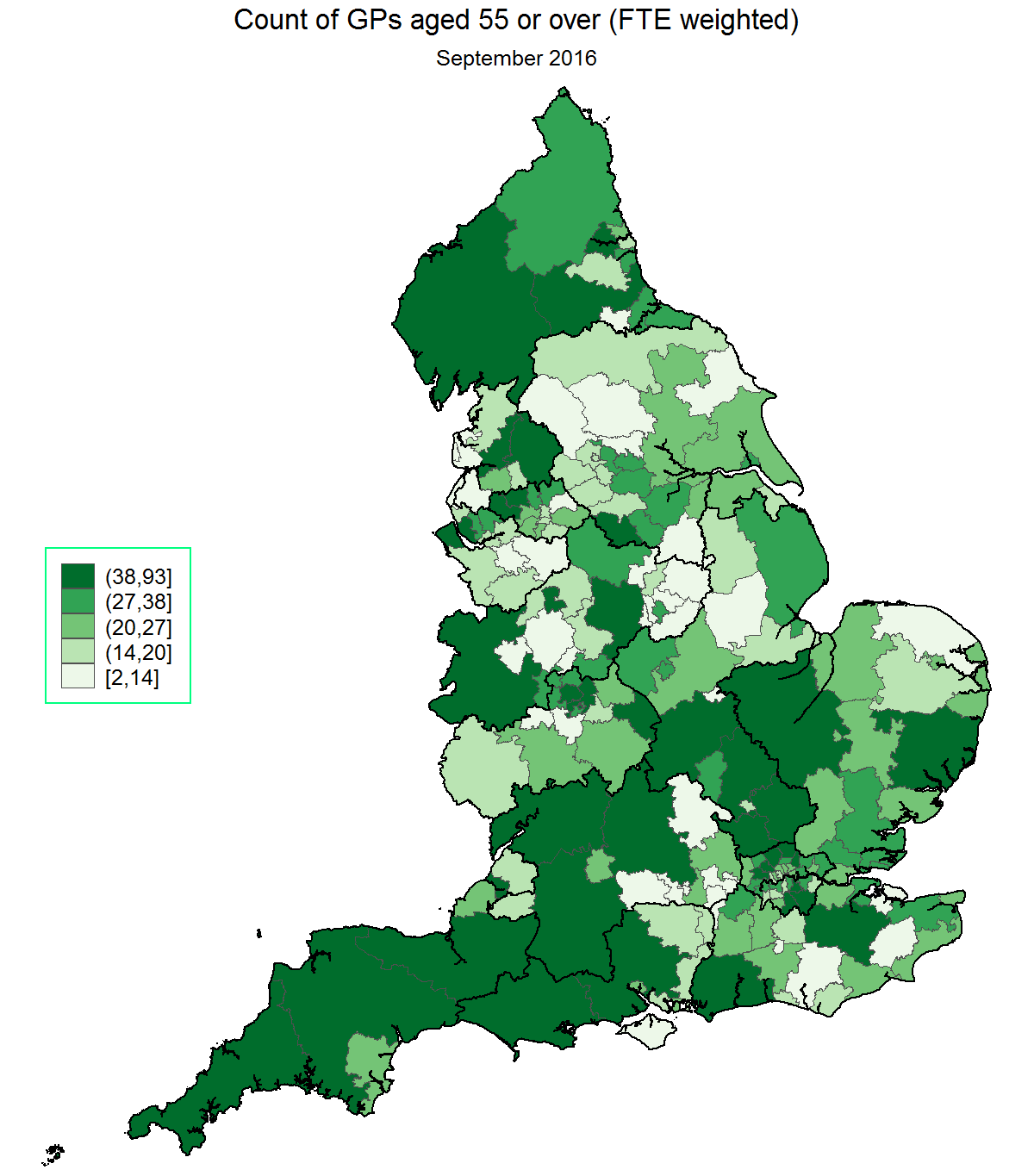


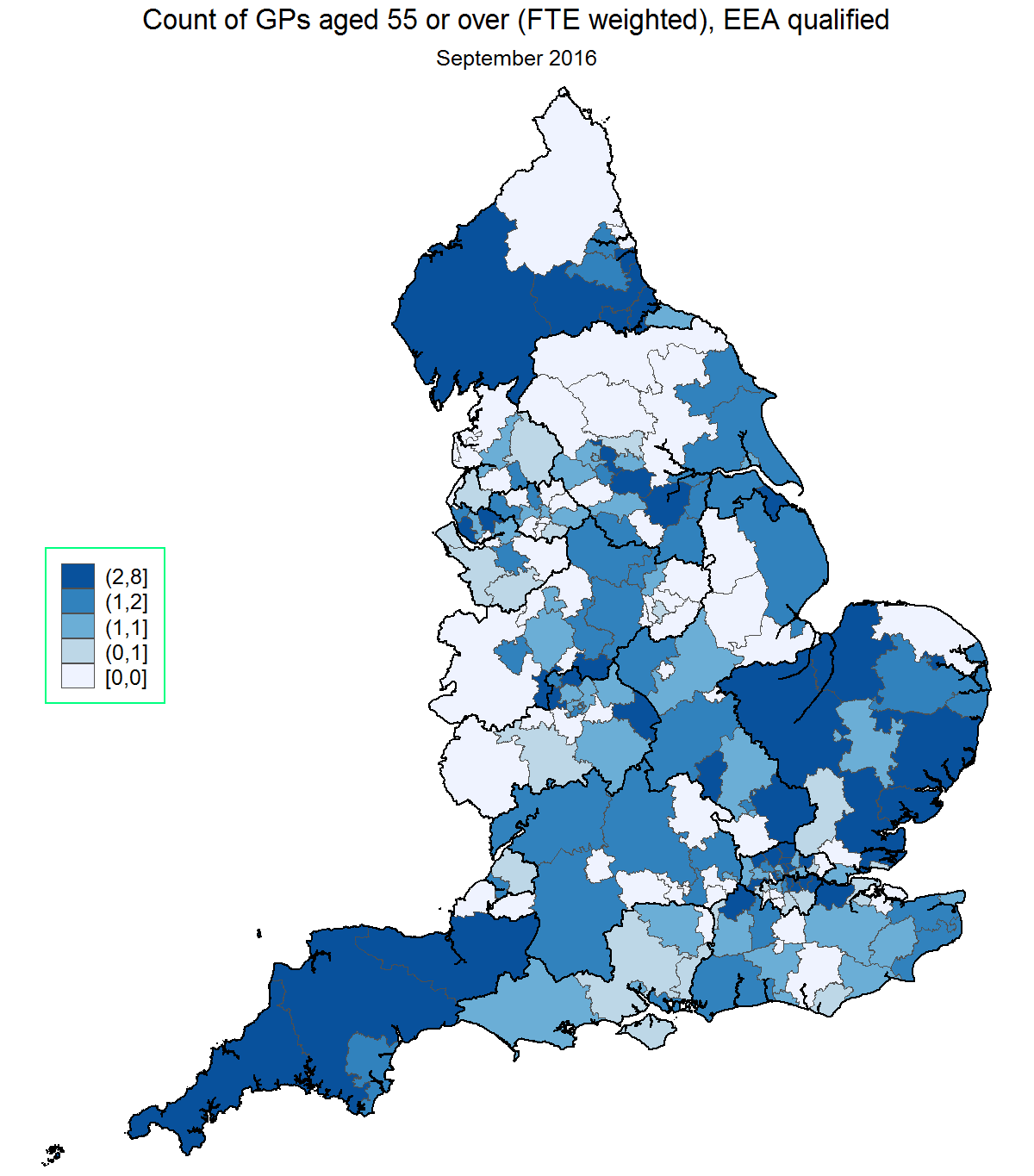


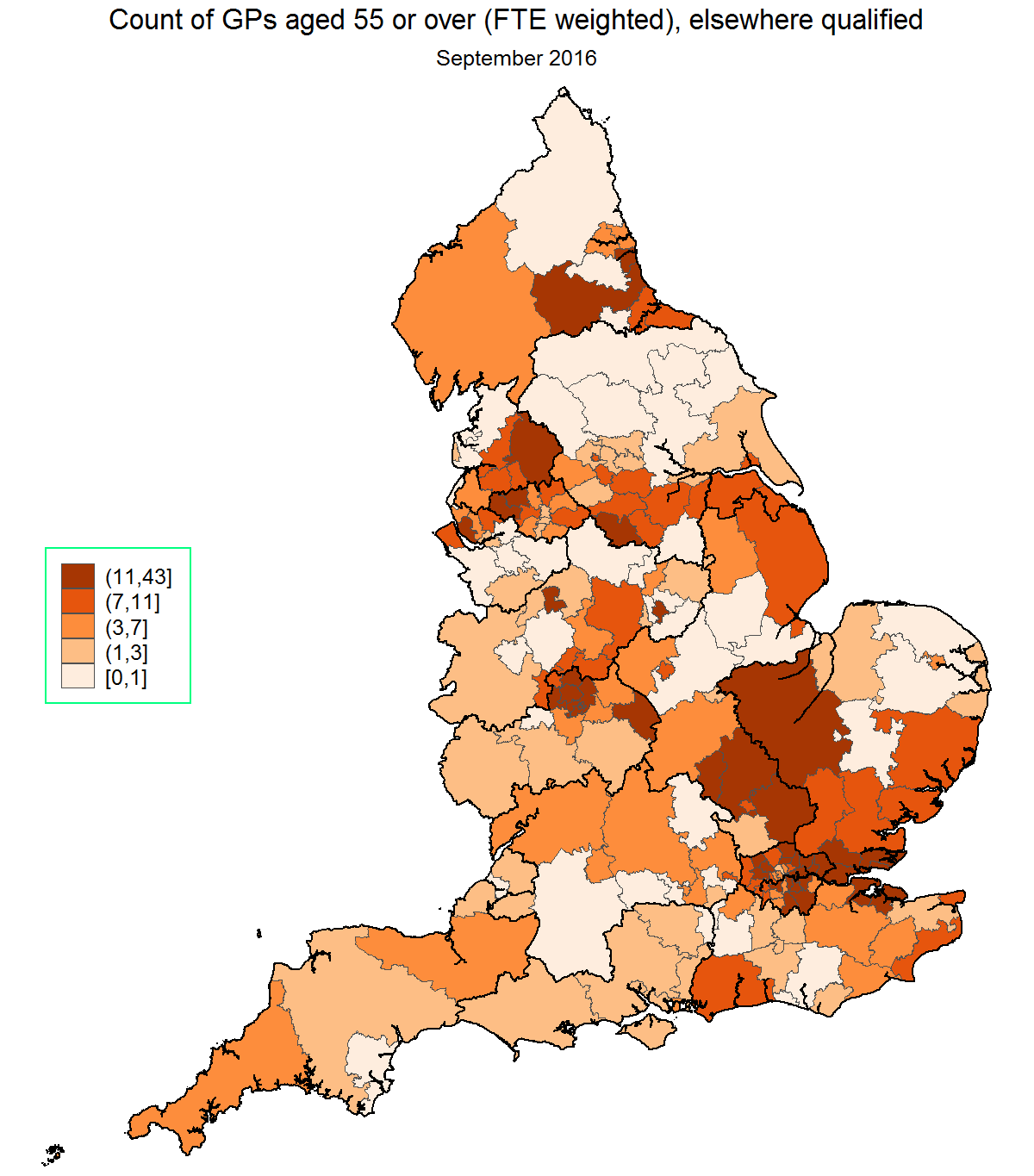


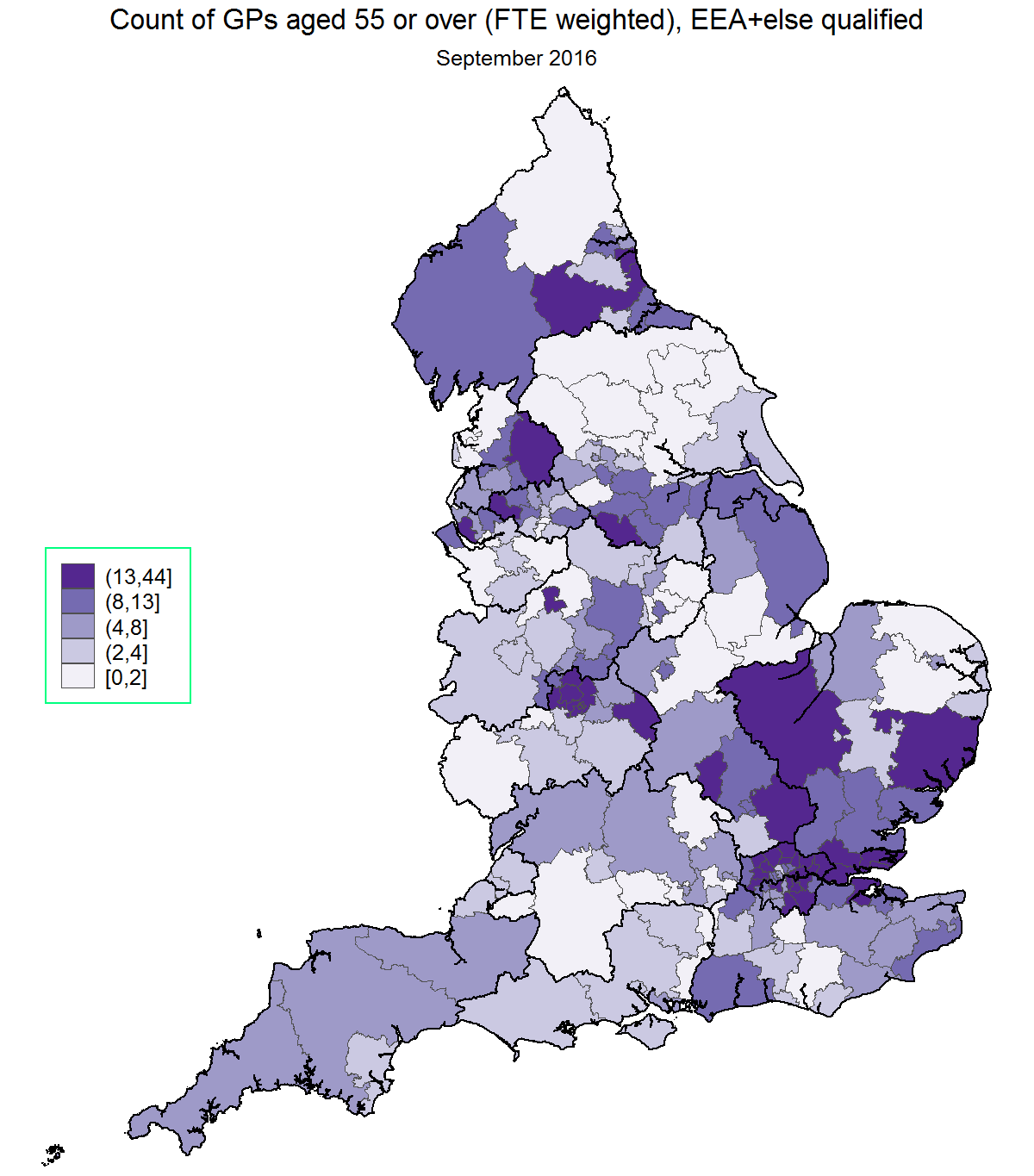


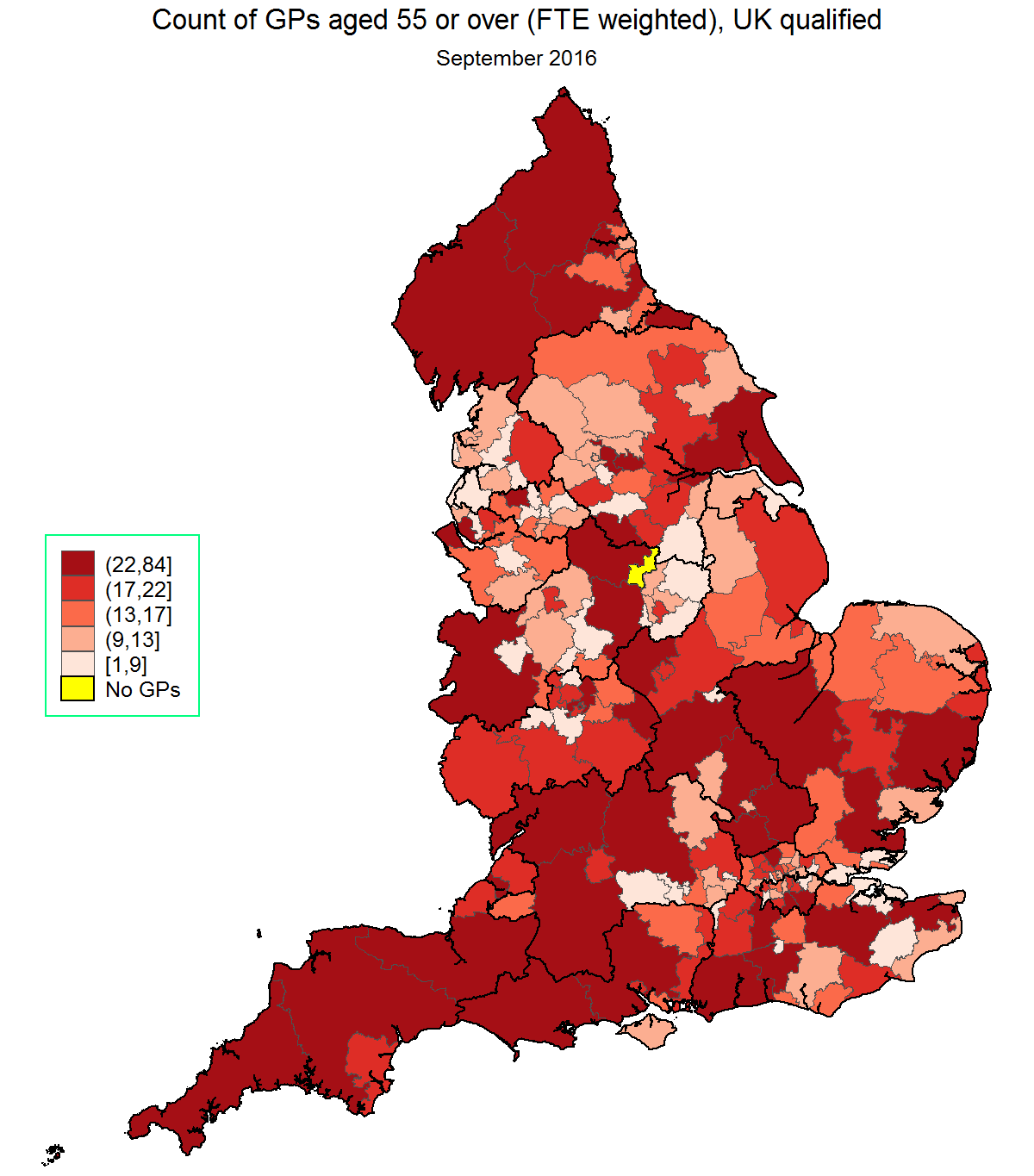


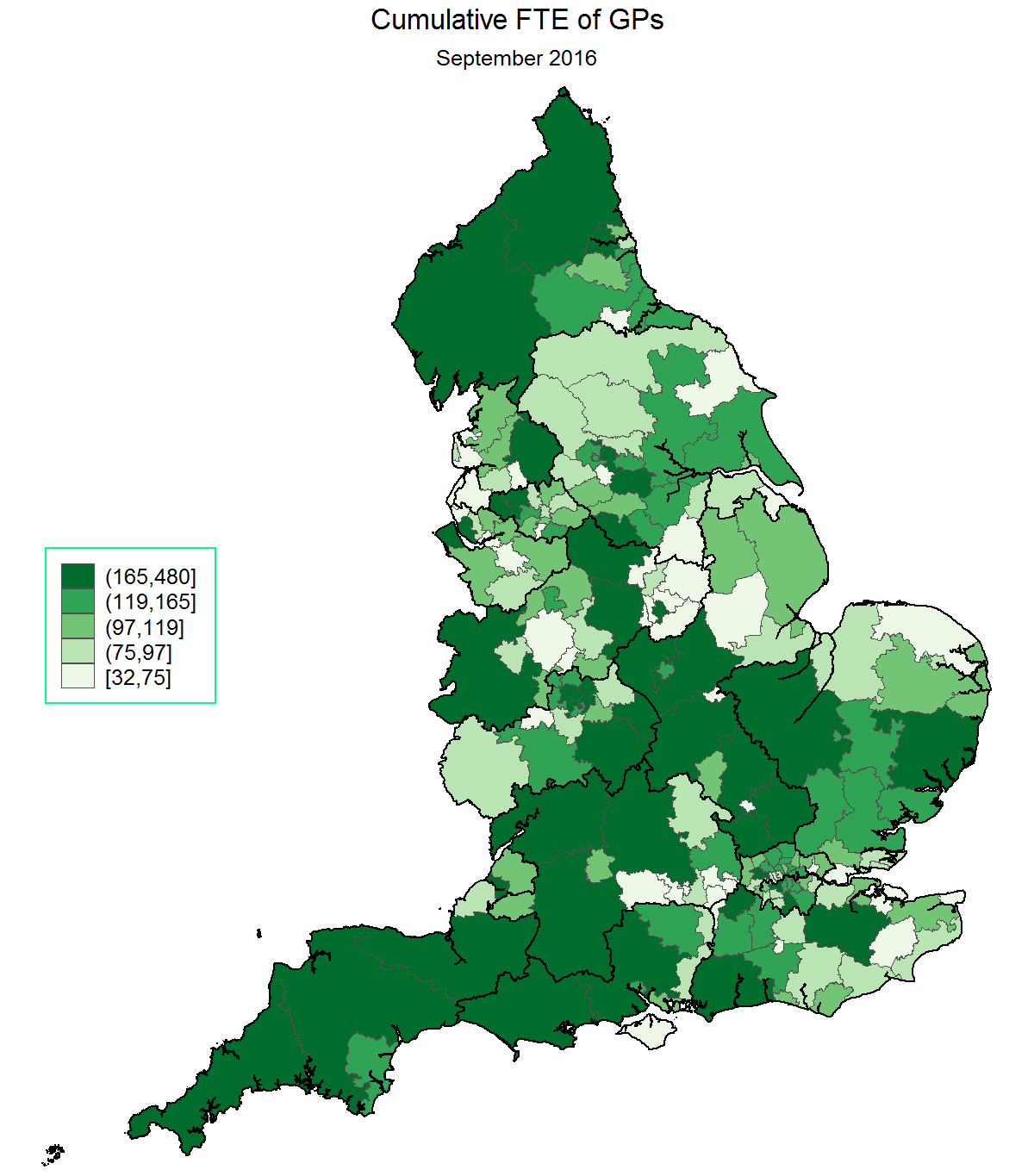


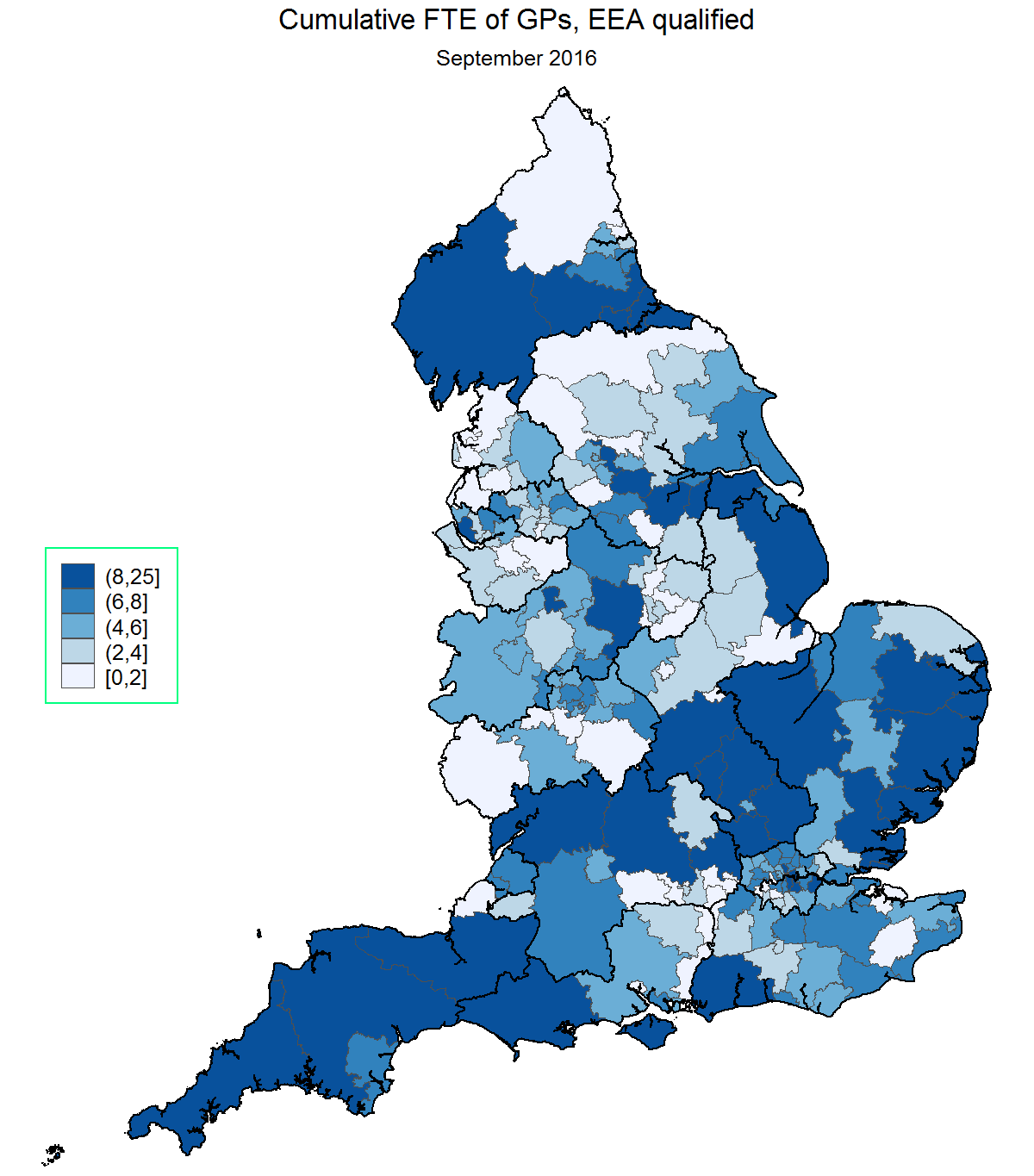


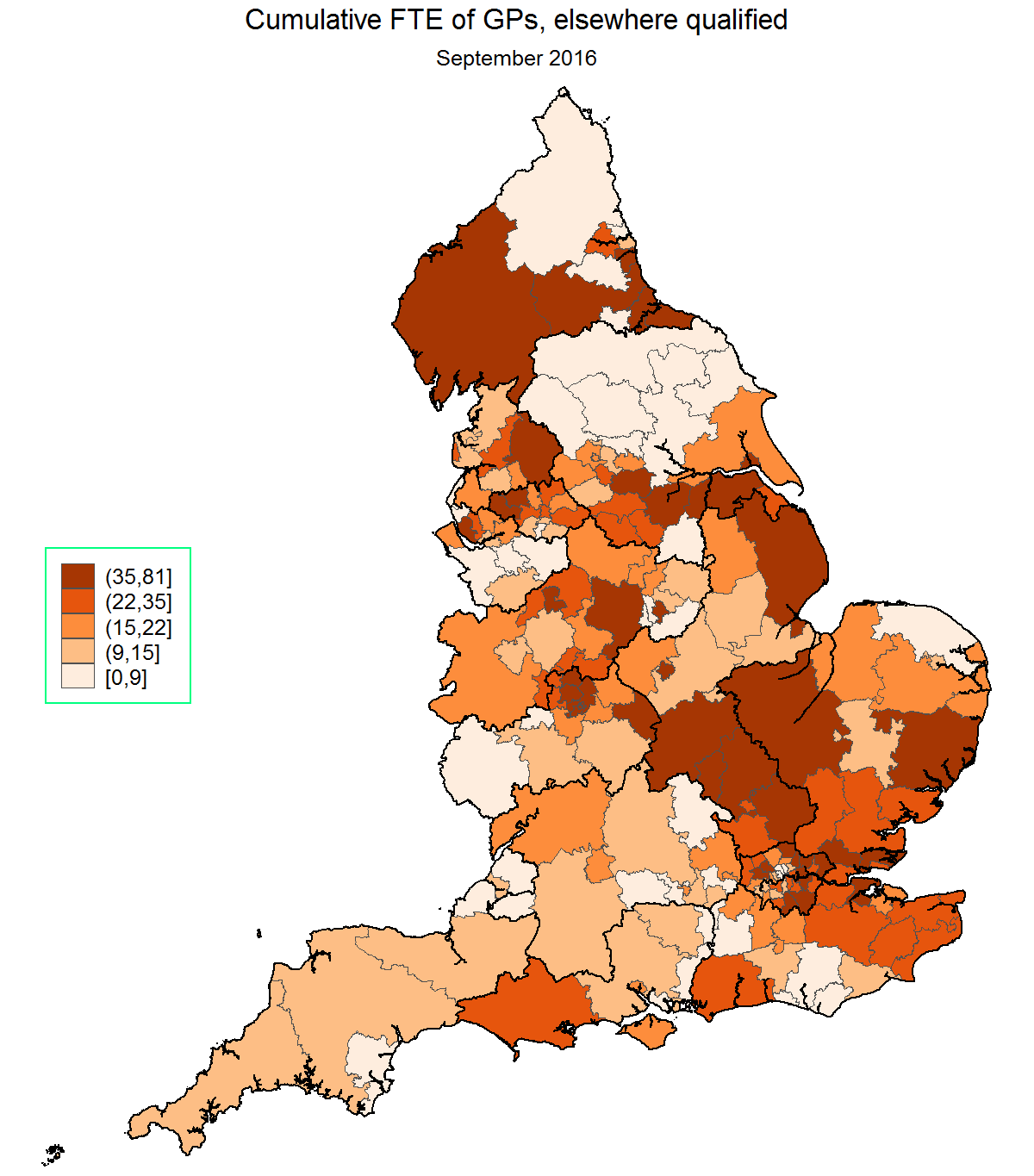


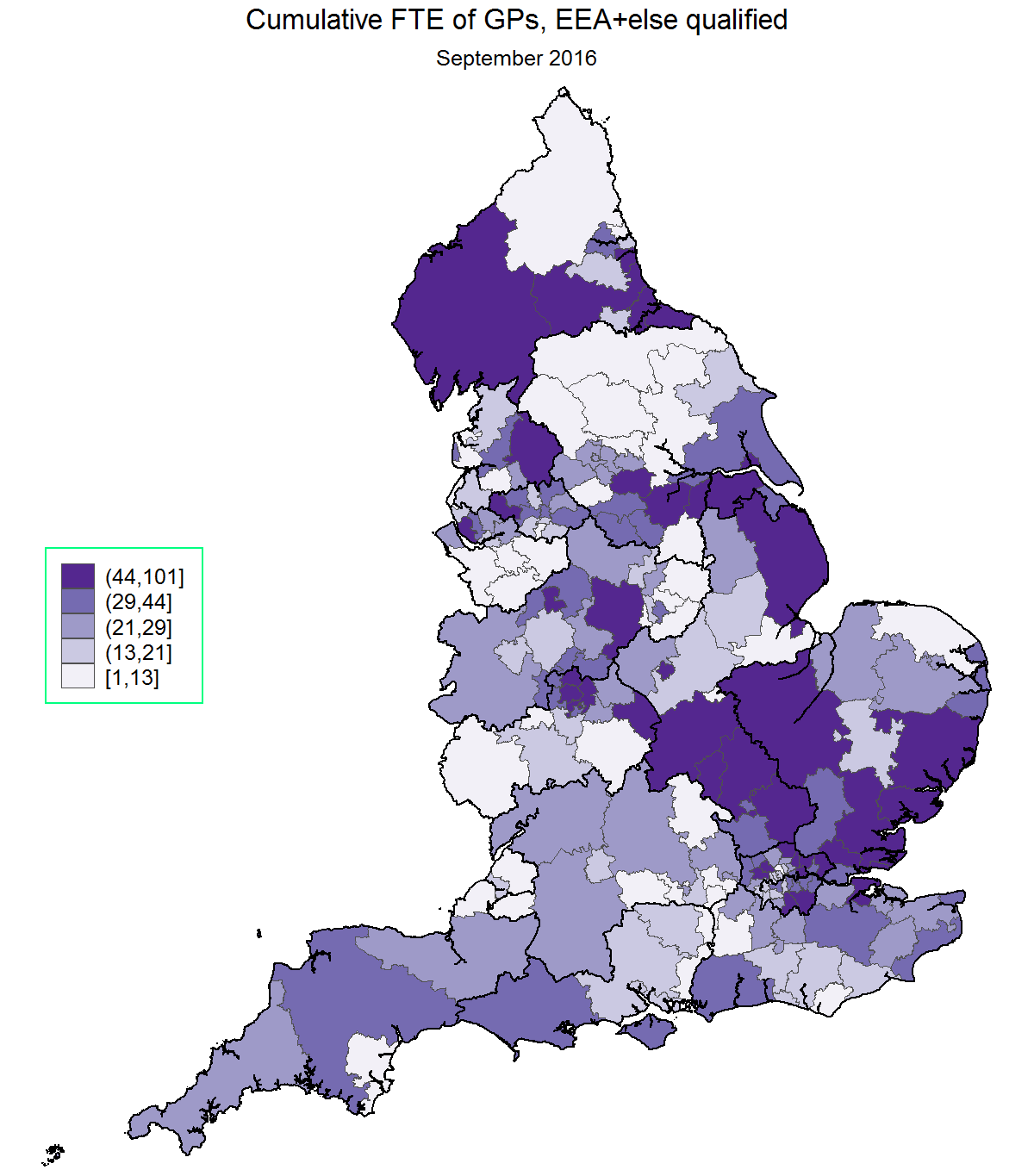


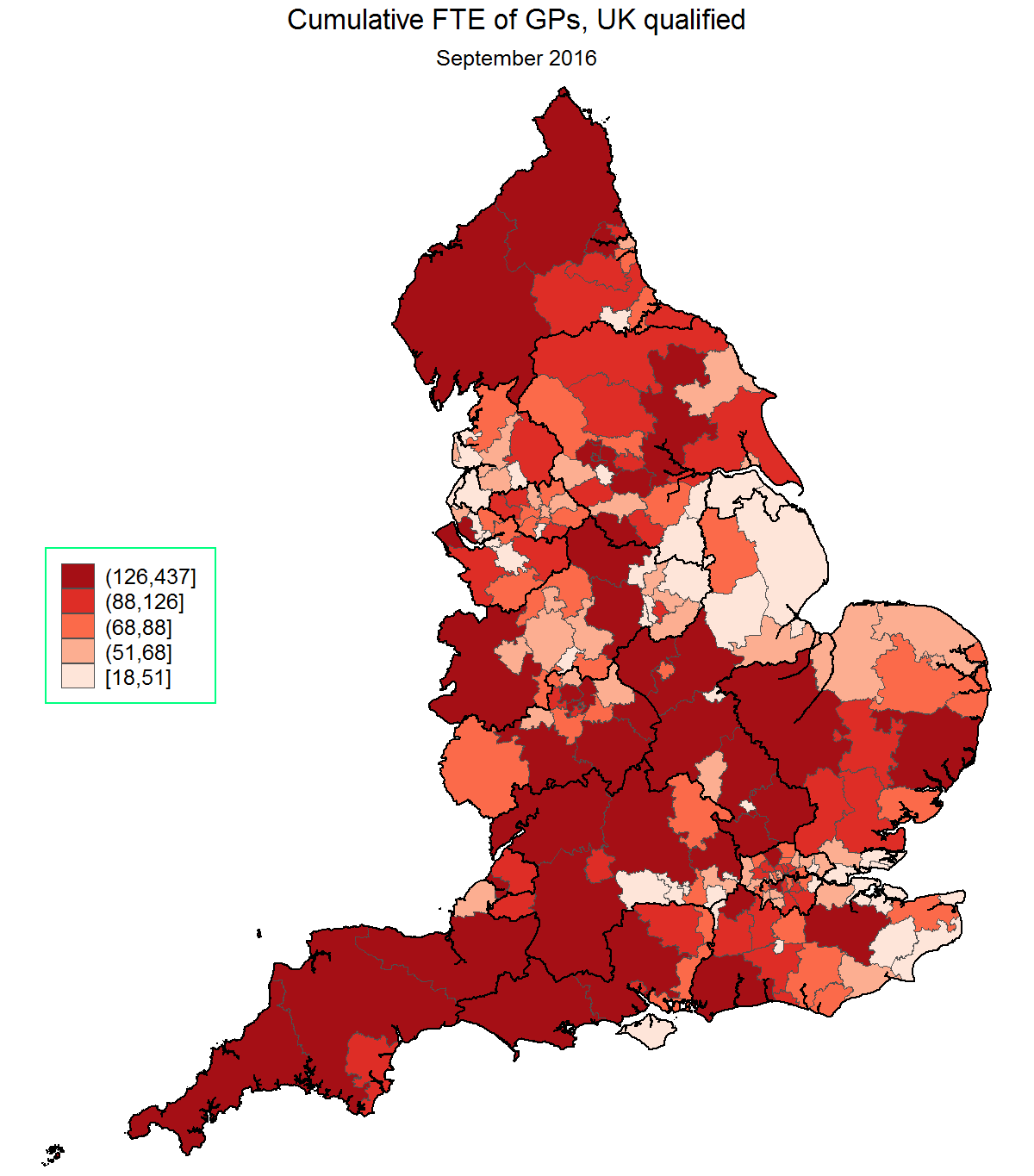


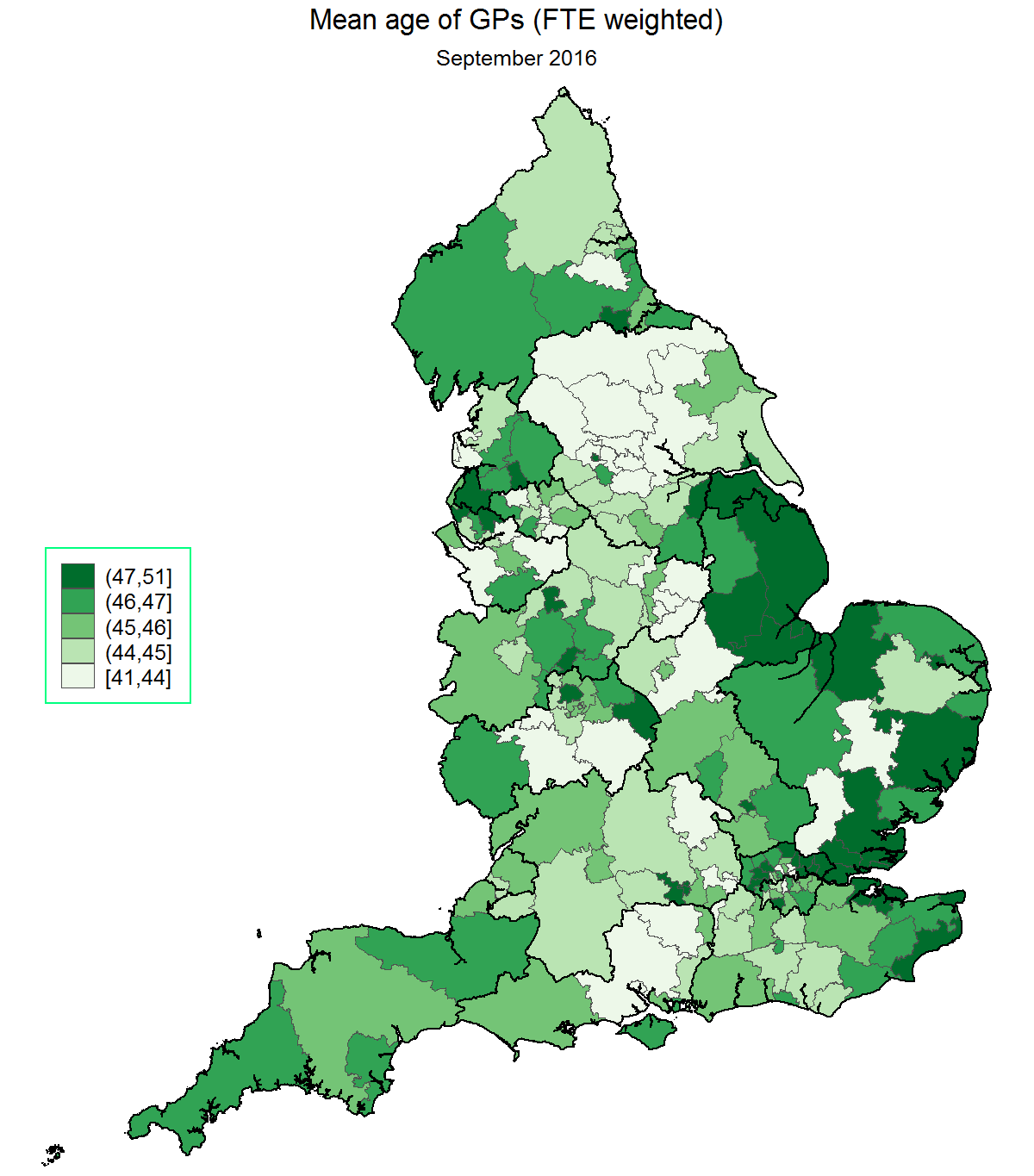


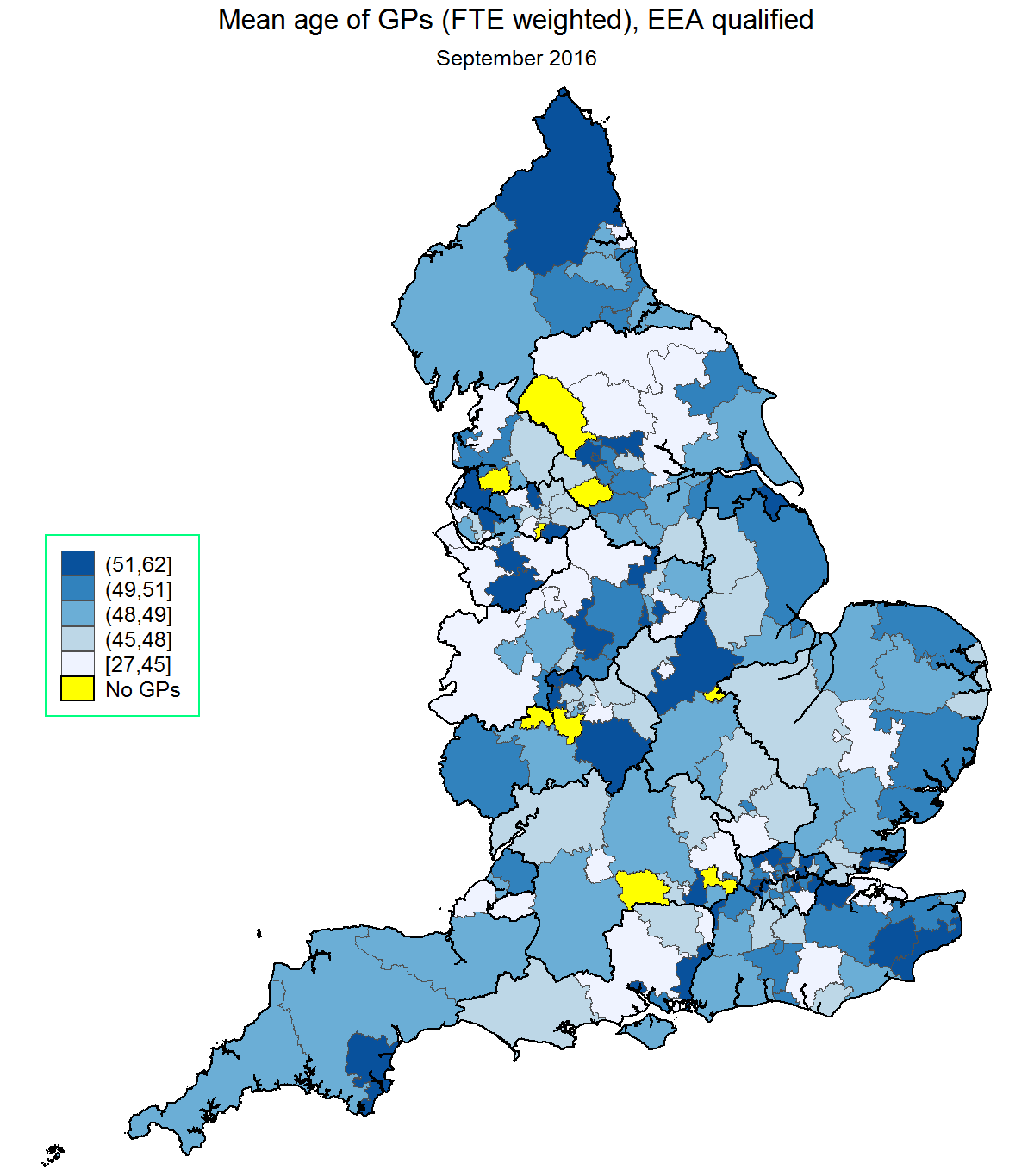


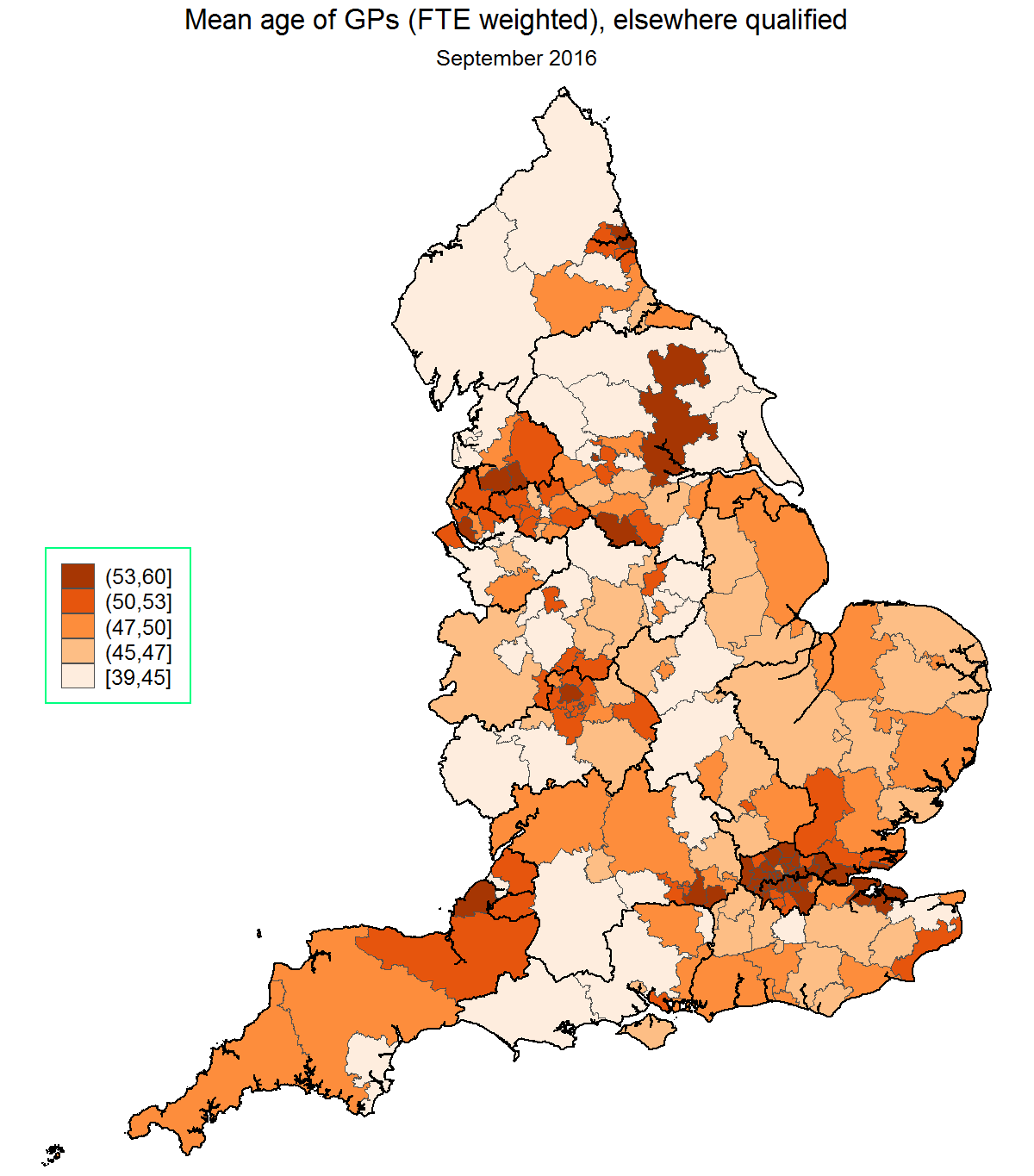


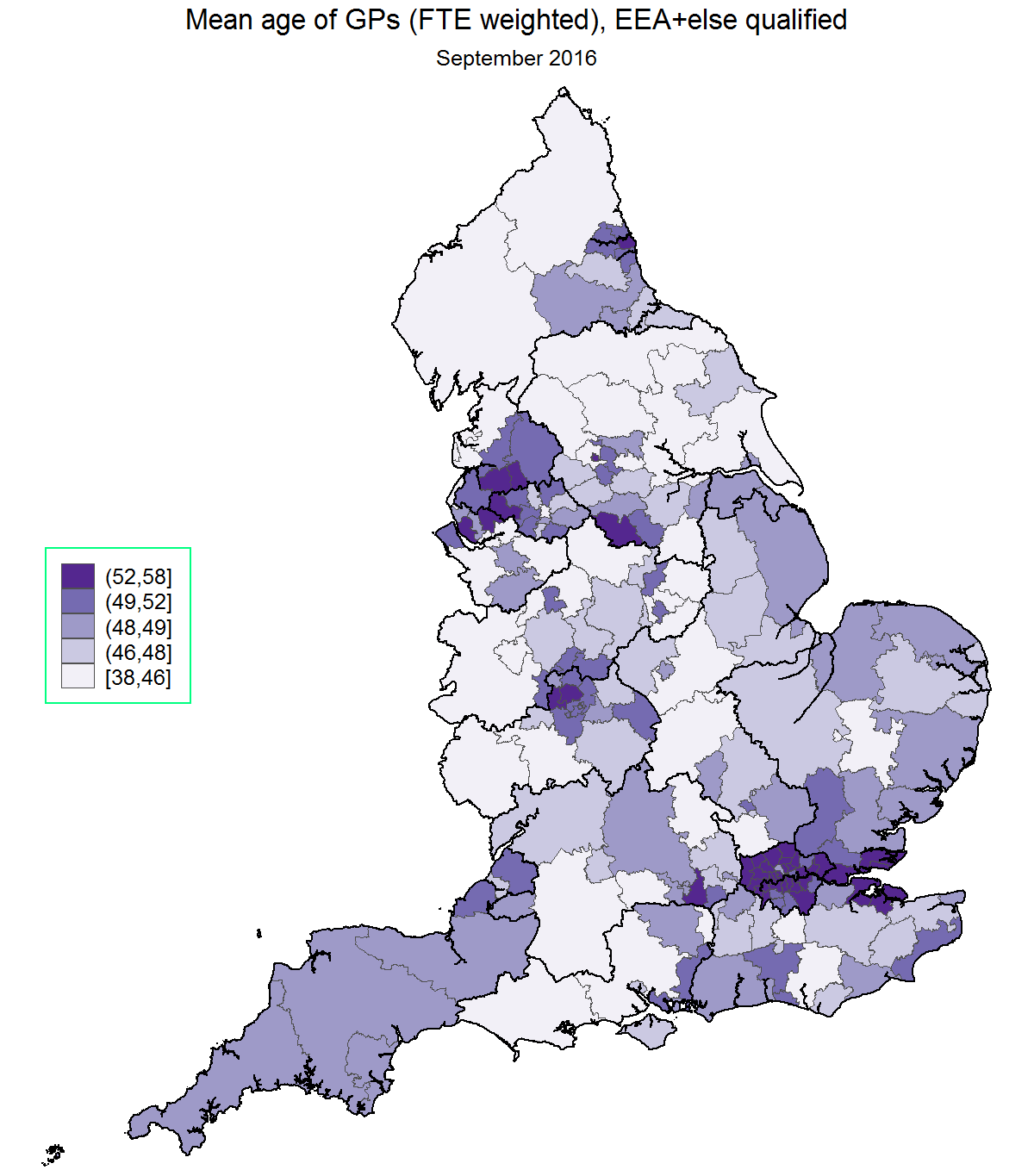


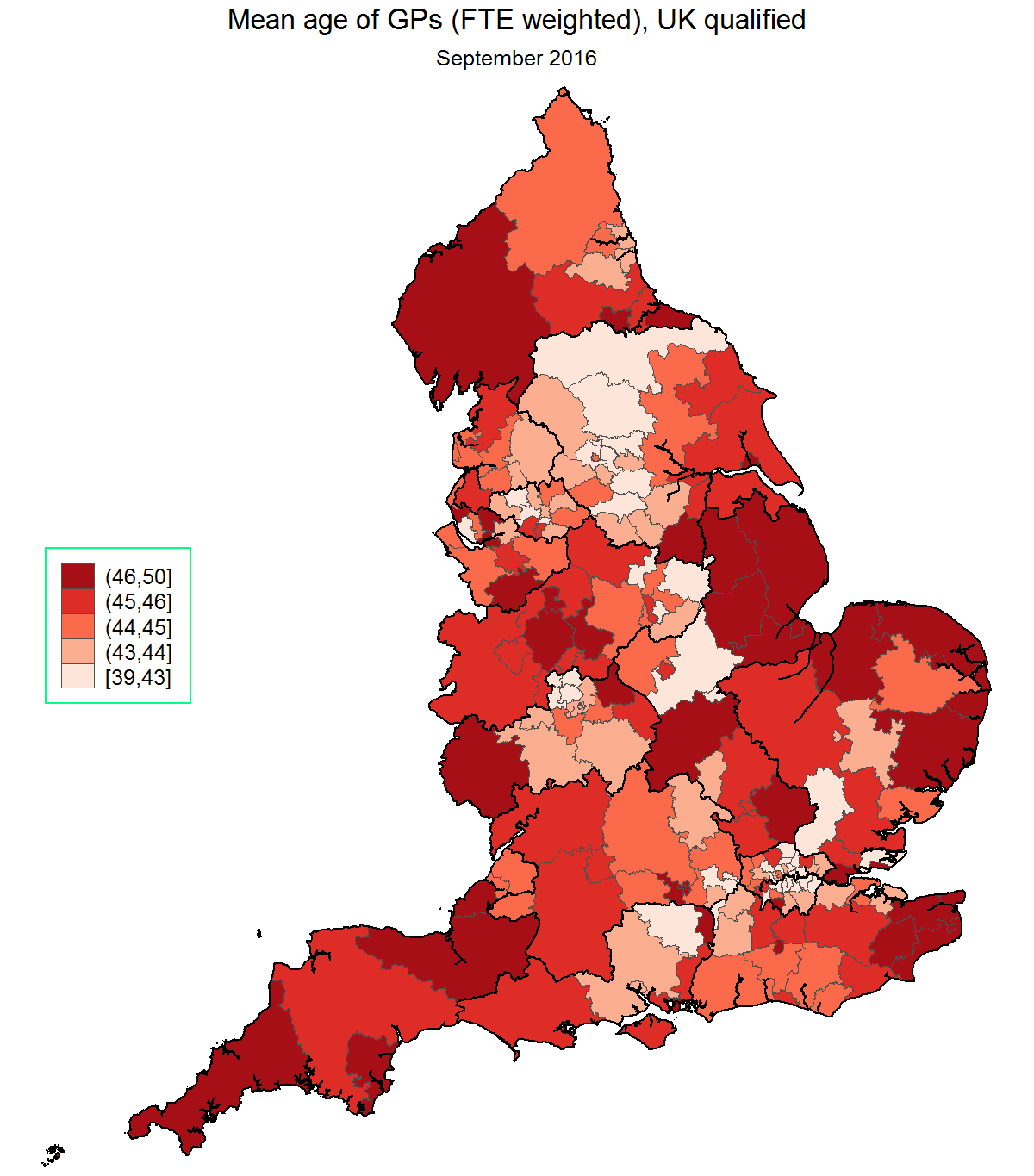


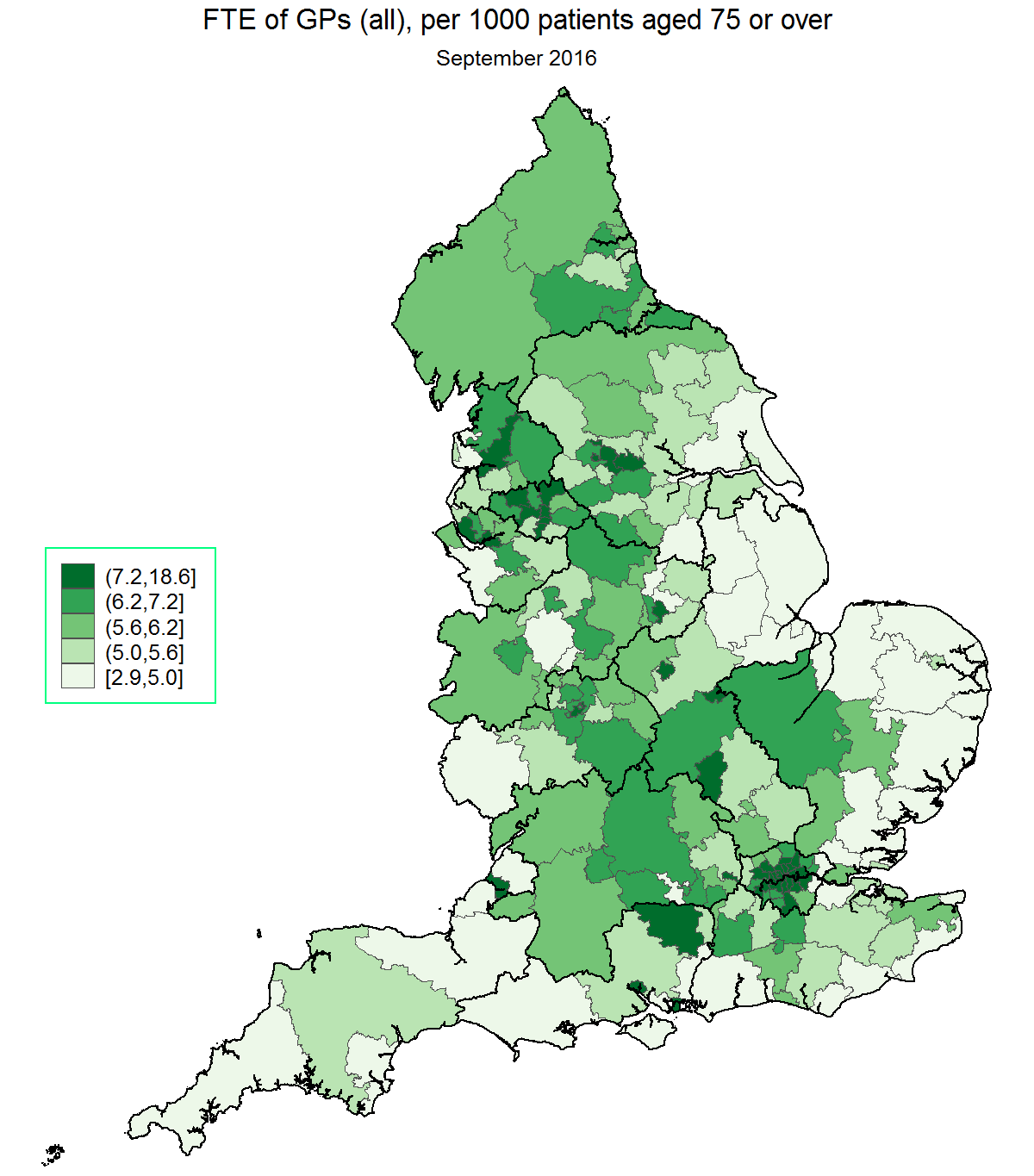


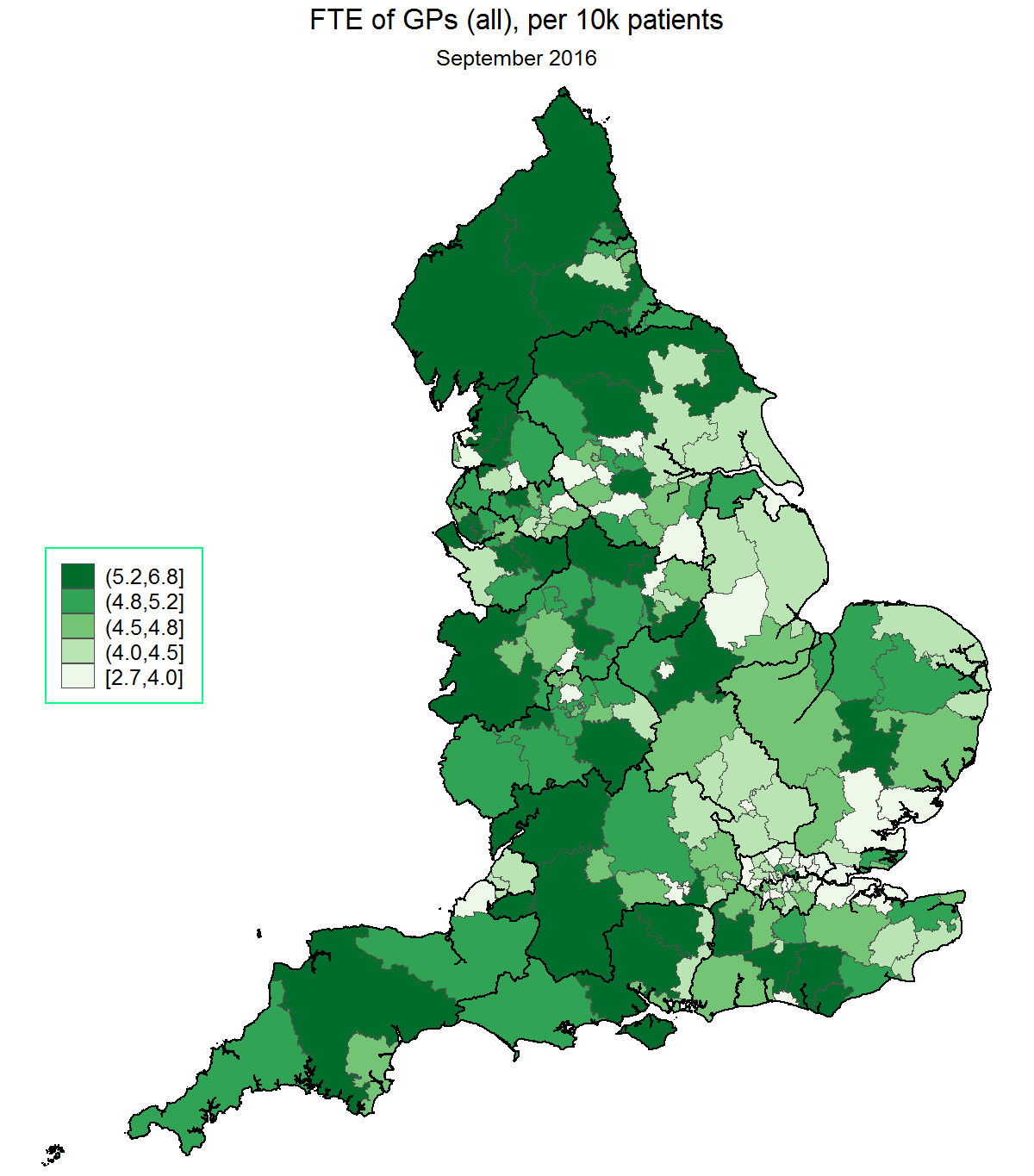


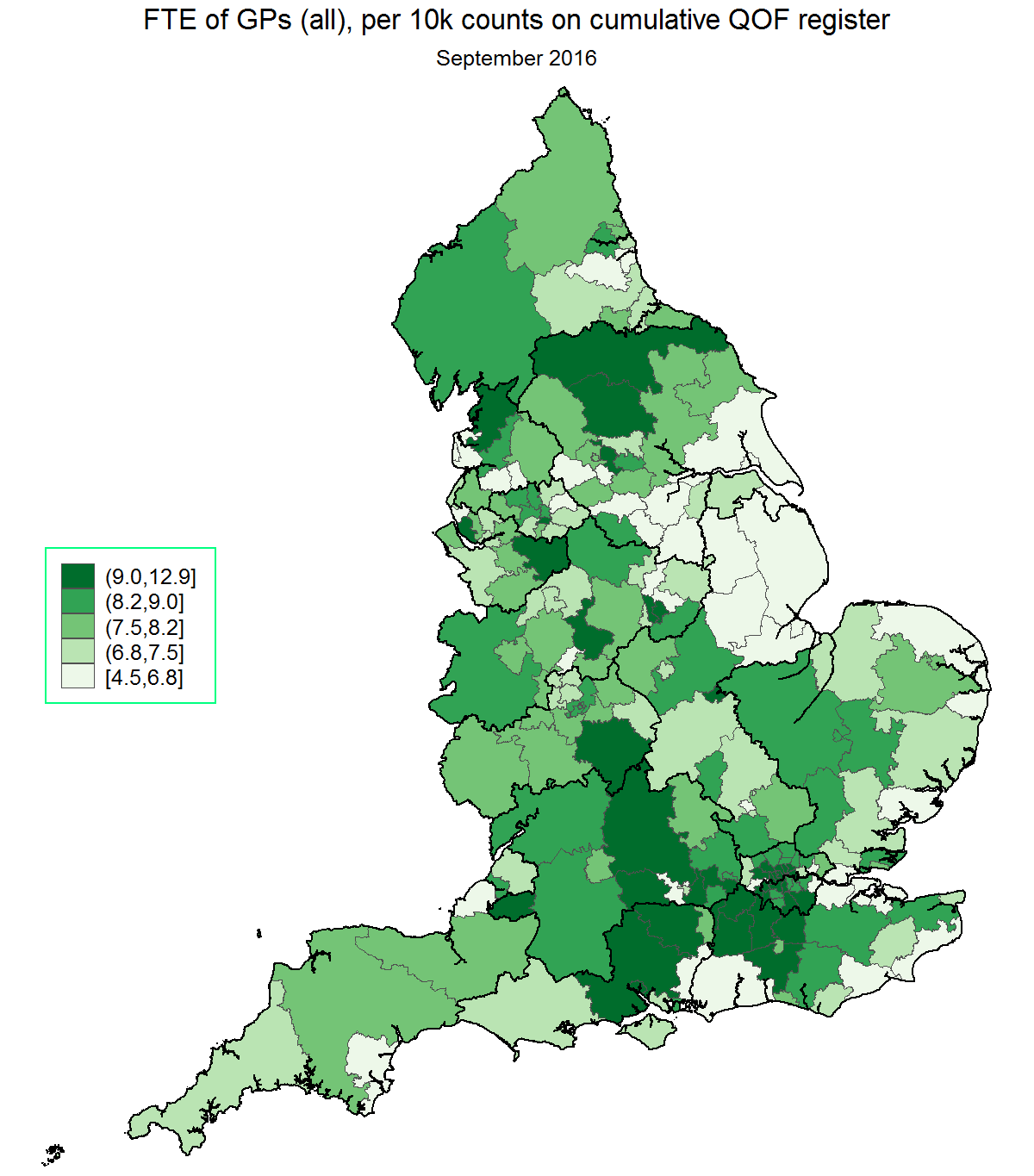


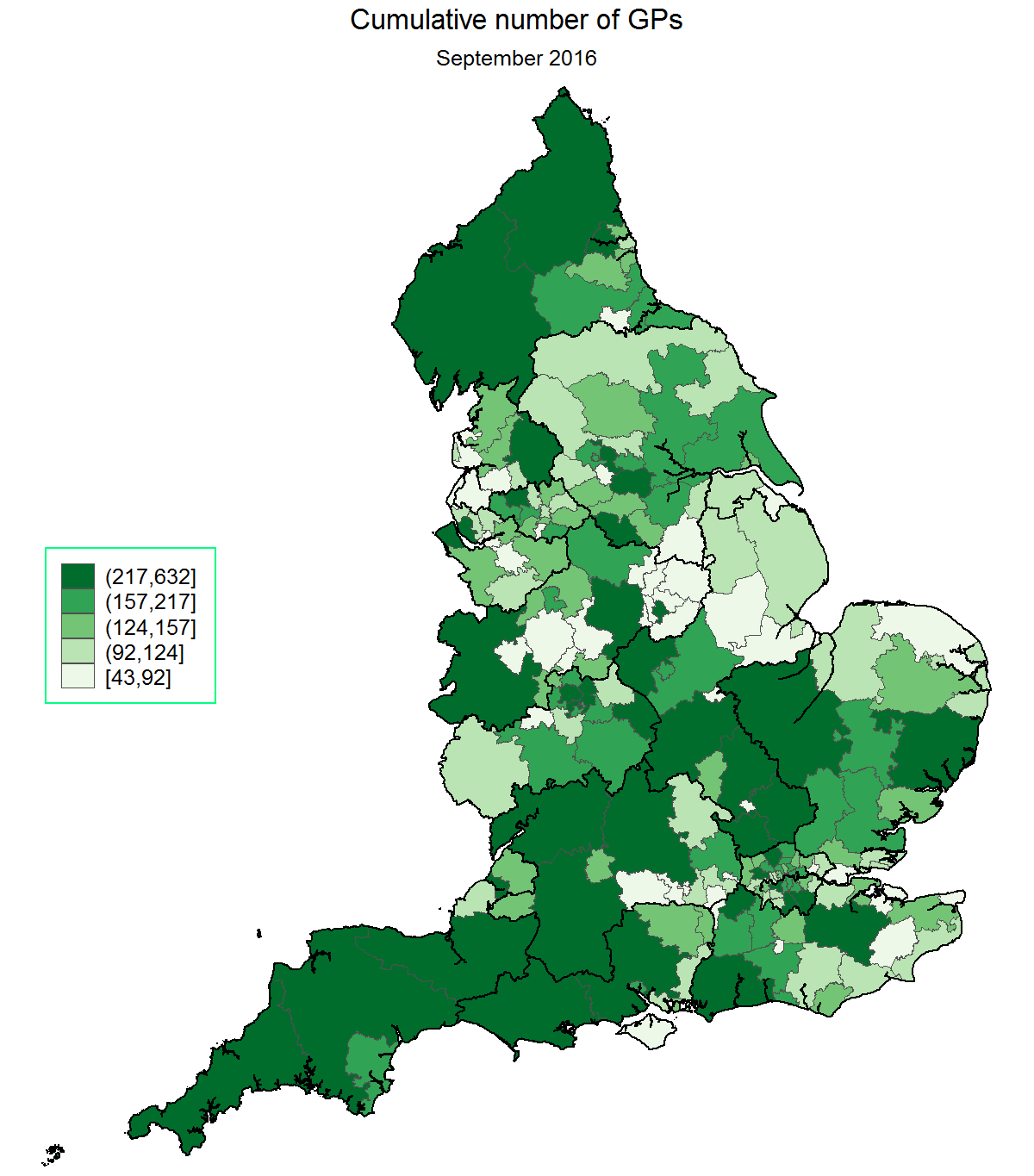


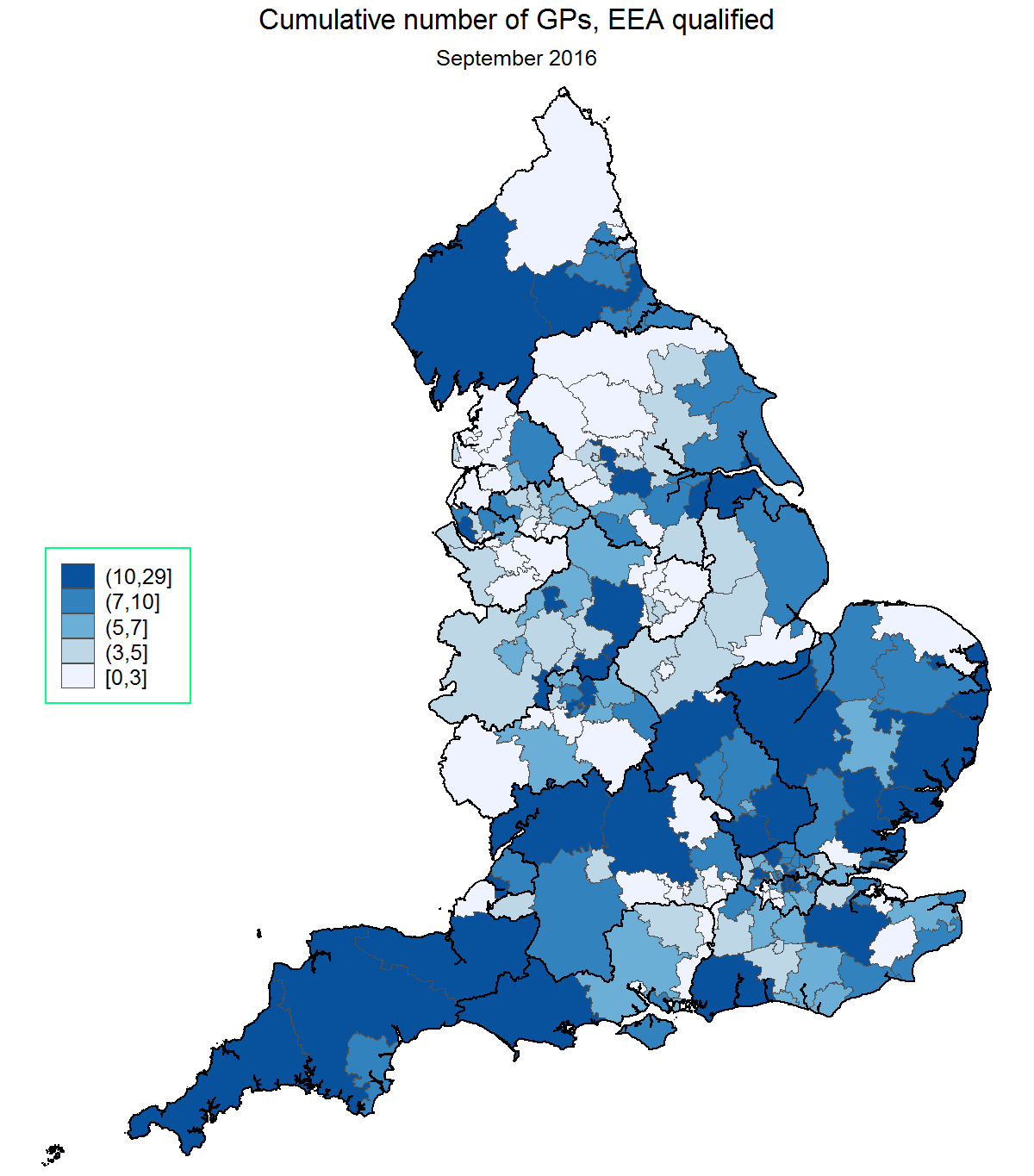


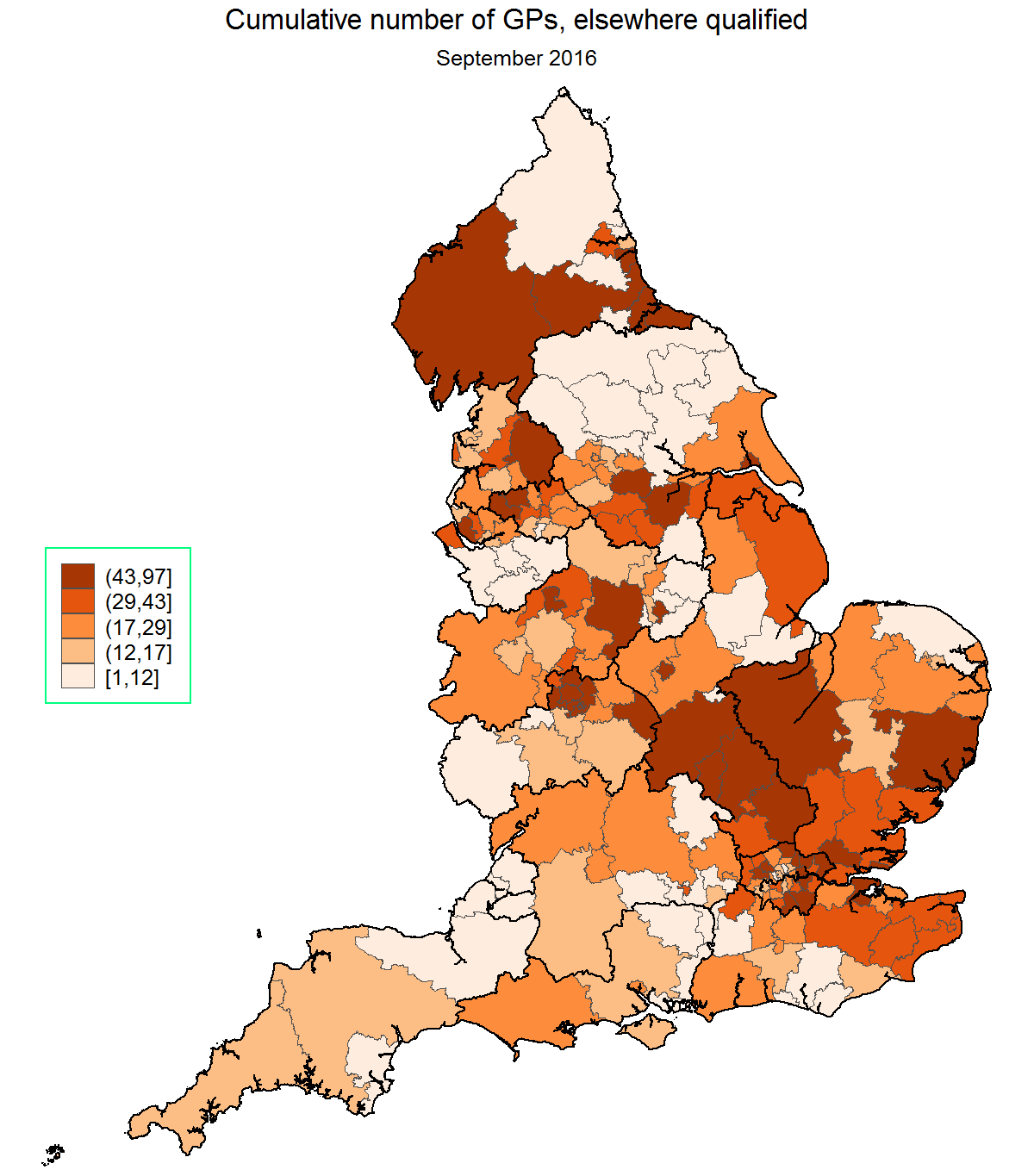


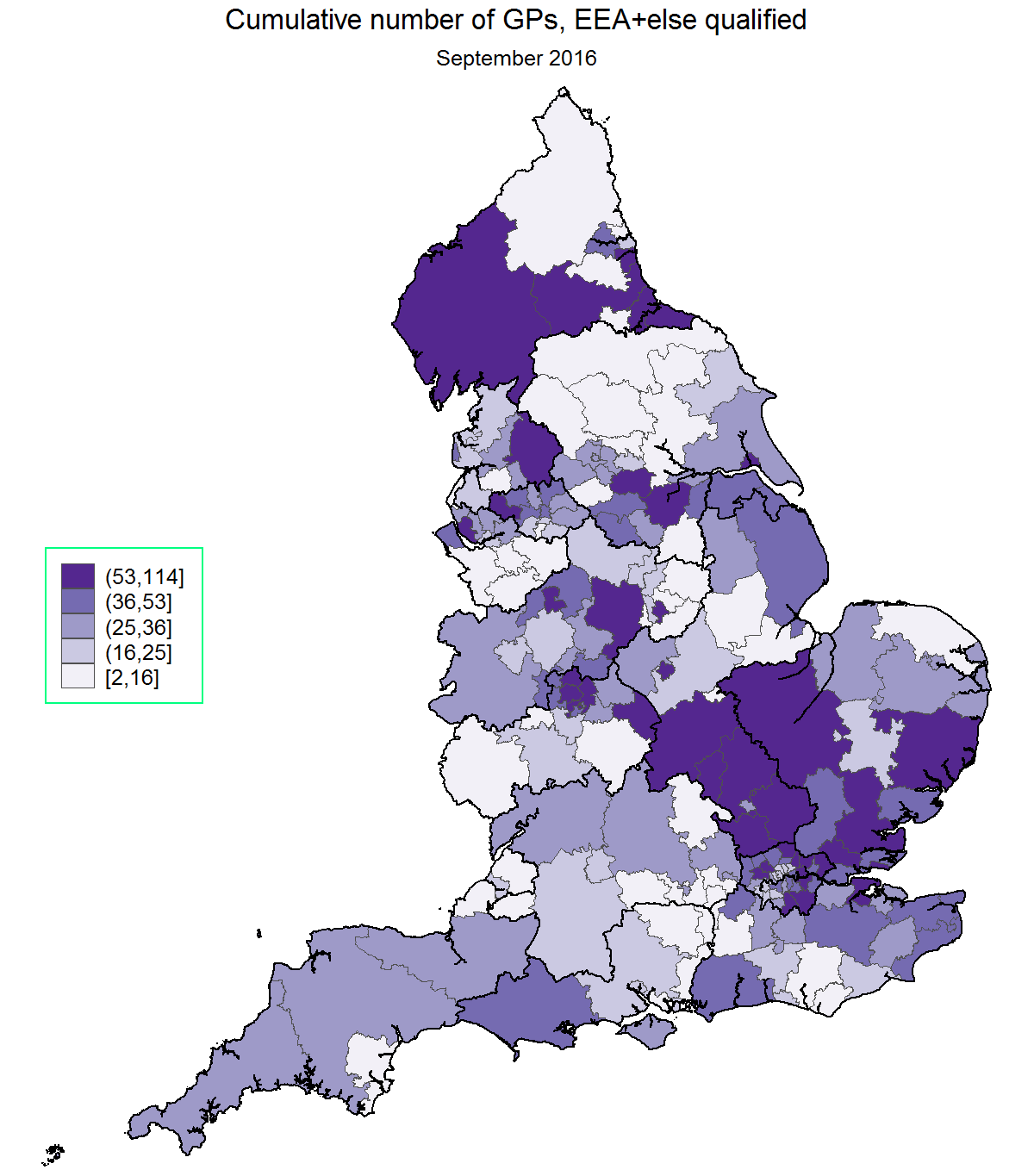


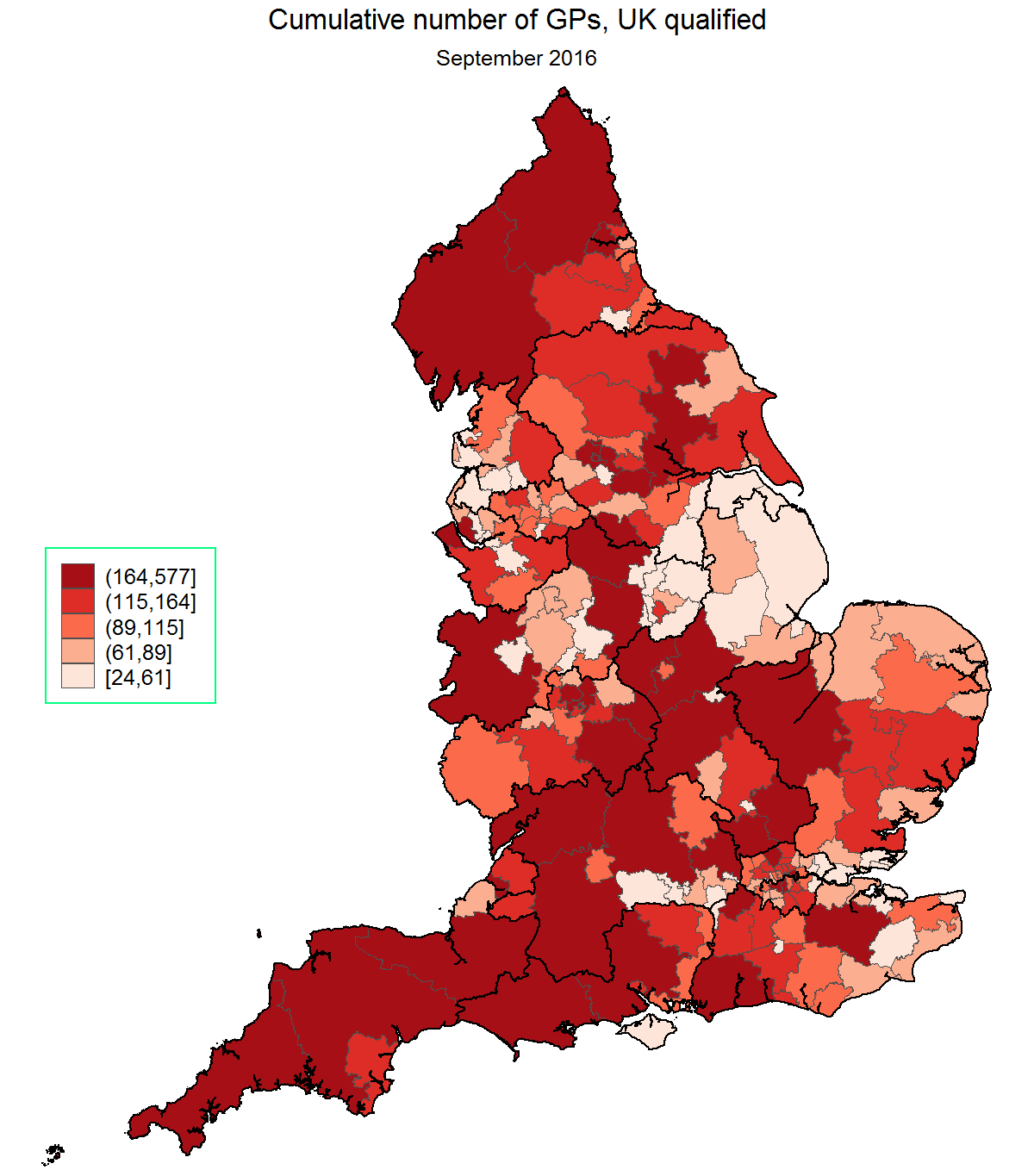

Supplement: Additional file 1: — Additional tables and spatial graphs. (DOCX 3450 kb) [file 12916_2017_953_MOESM1_ESM.docx]
